# Supplementary figures and images for: The fate of pyruvate dictates cell growth by modulating cellular redox potential
Source: eLife. 2025 Dec 16;13:RP103705. doi: 10.7554/eLife.103705 (PMC12707817; doi:10.7554/eLife.103705)

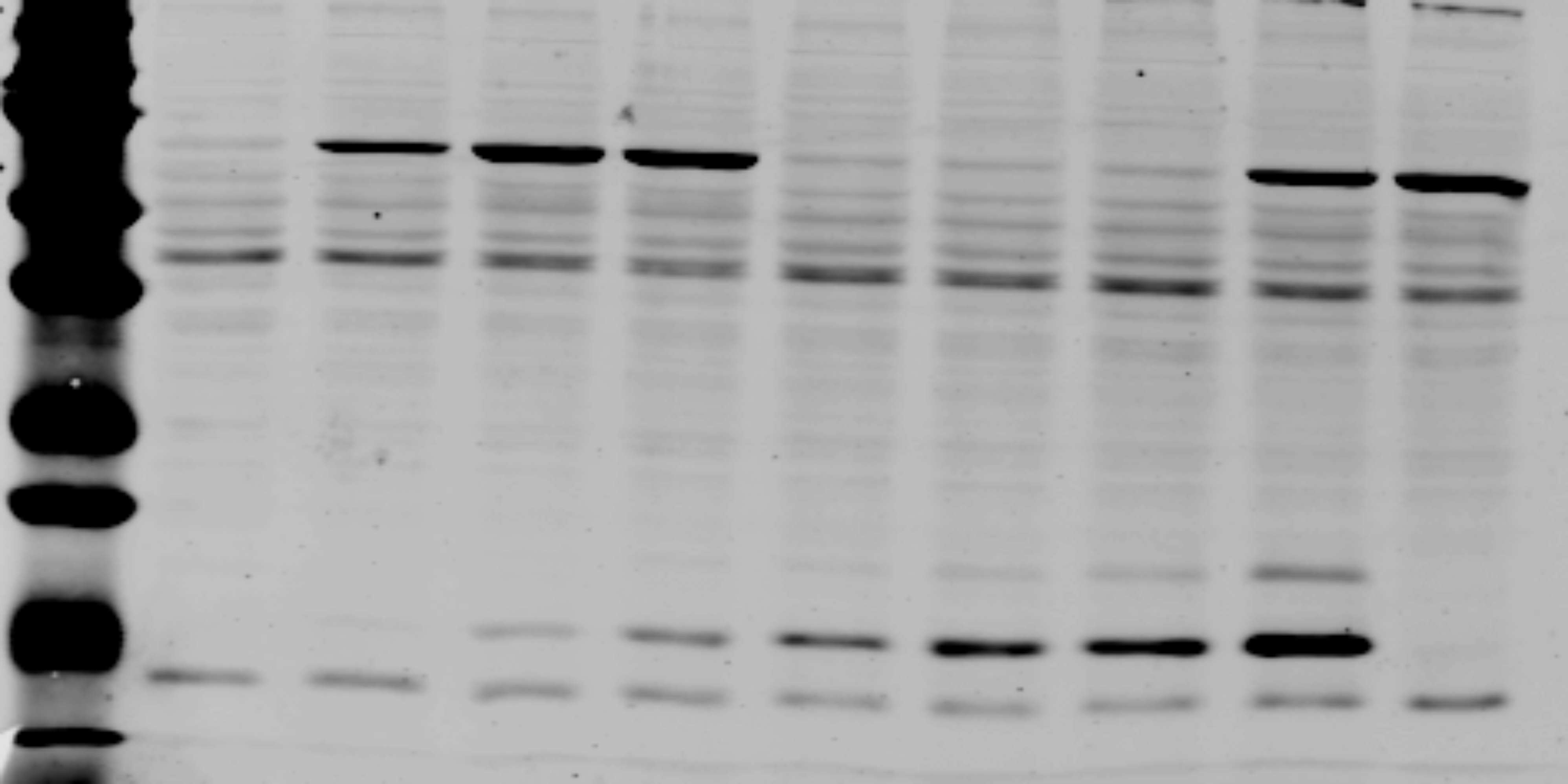

Supplement: Figure 3—source data 2. [file elife-103705-fig3-data2.zip › Figure 3-source data 4-Original files for western blot analysis displayed in Figure 3a/Original files for western blot analysis displayed in Figure 3a 2.tif]

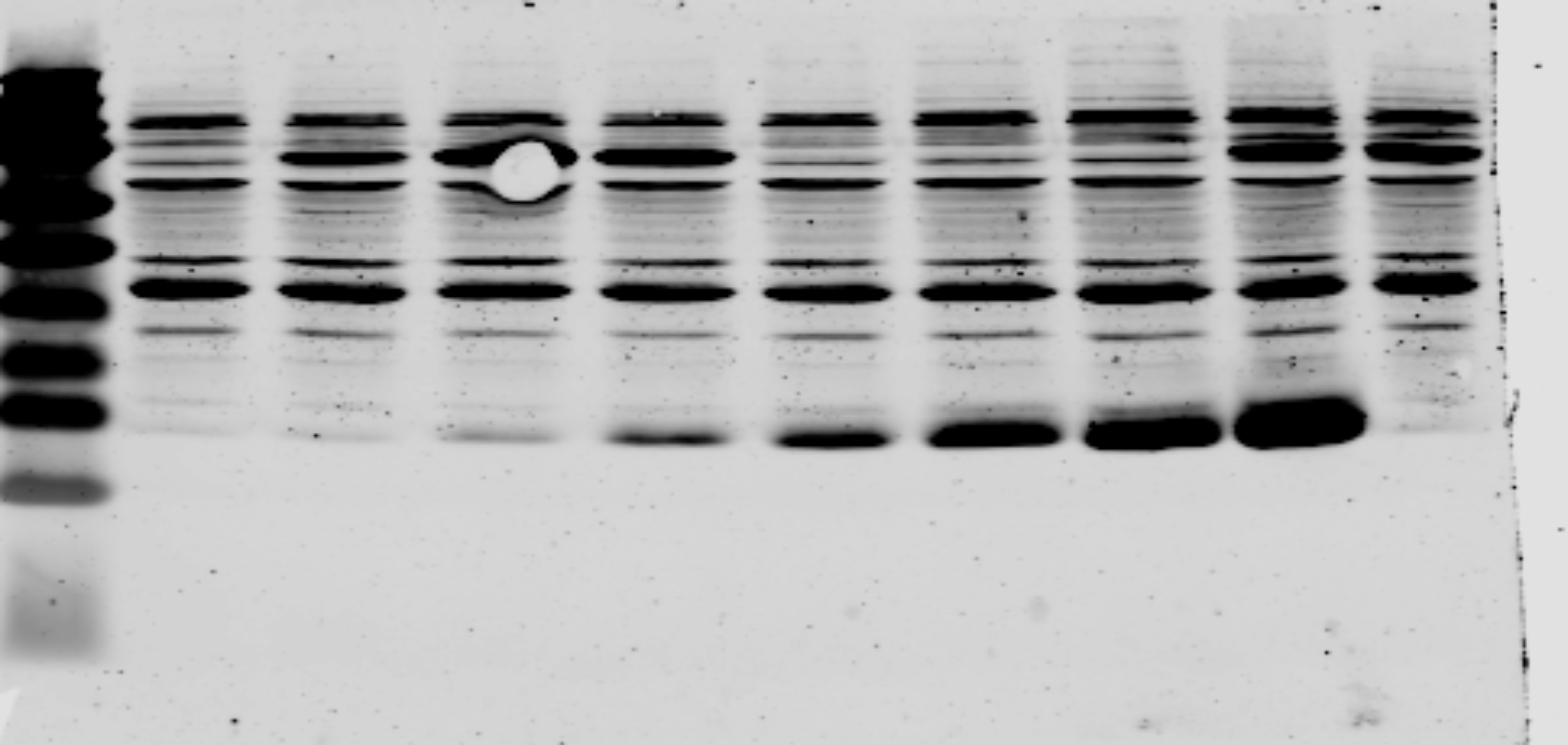

Supplement: Figure 3—source data 2. [file elife-103705-fig3-data2.zip › Figure 3-source data 4-Original files for western blot analysis displayed in Figure 3a/Original files for western blot analysis displayed in Figure 3a 3.tif]

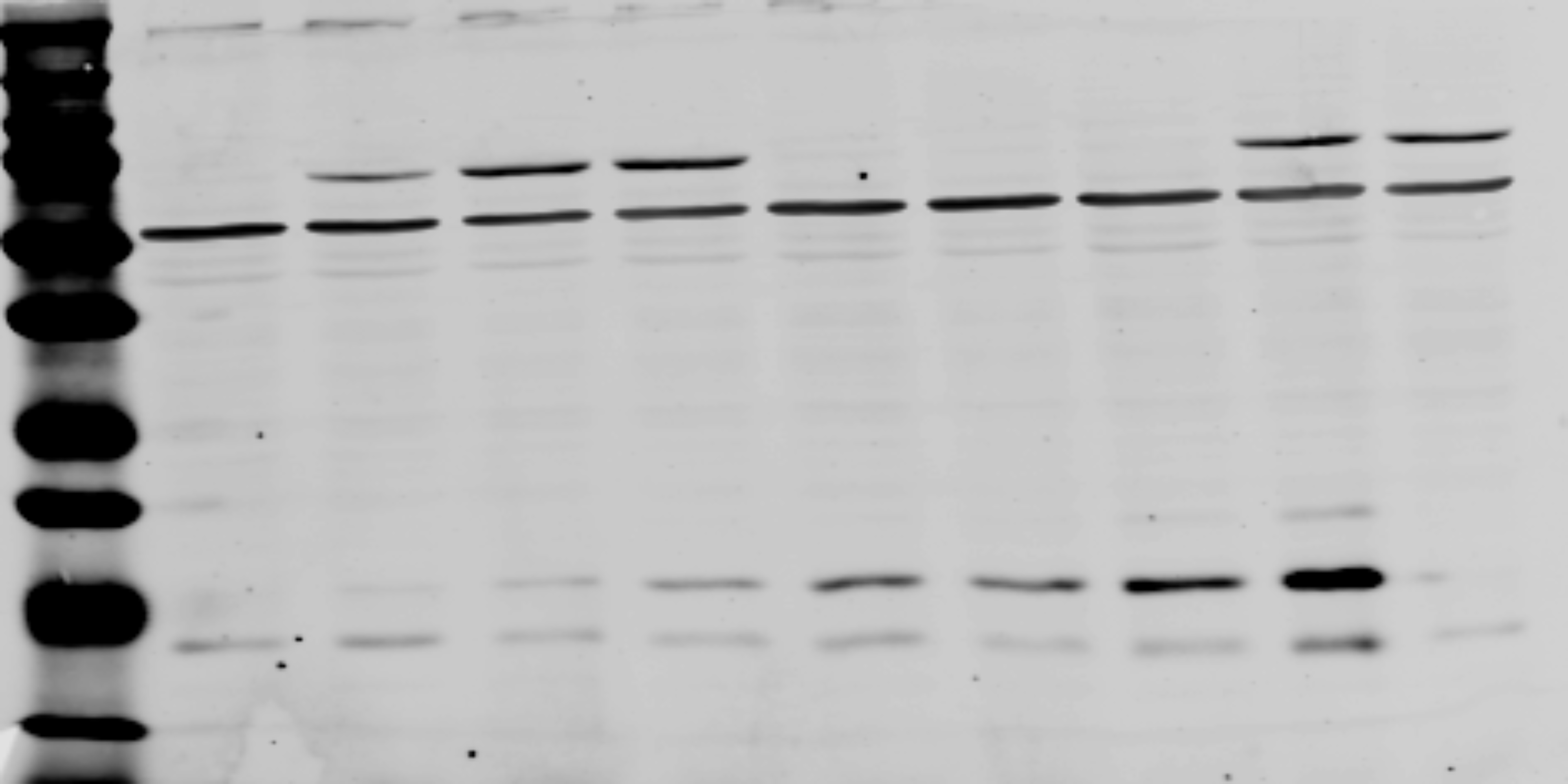

Supplement: Figure 3—source data 2. [file elife-103705-fig3-data2.zip › Figure 3-source data 4-Original files for western blot analysis displayed in Figure 3a/Original files for western blot analysis displayed in Figure 3a 1.tif]

0 2 4 6 8 10 12 24 EV24 DOX (Hours)

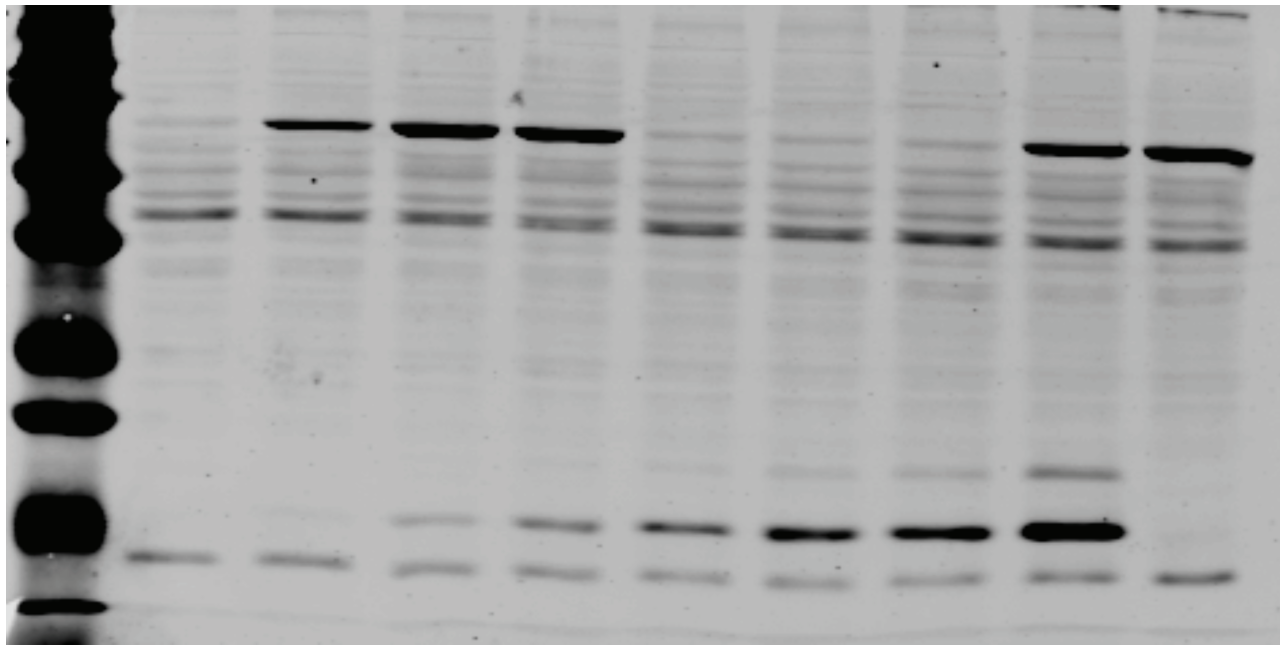

Citrate Synthase

MPC2-HA  
Endogenous MPC2

Supplement: Figure 3—source data 3. [file elife-103705-fig3-data3.zip › Figure 3-source data 2- PDF files containing originall western blots for Figure 3a,indicating the relevant bands and treatments./Source data Figure 3a uncropped western blots 2.pdf]

0

2

4

6

8

10

12

24

EV24 DOX (Hours)

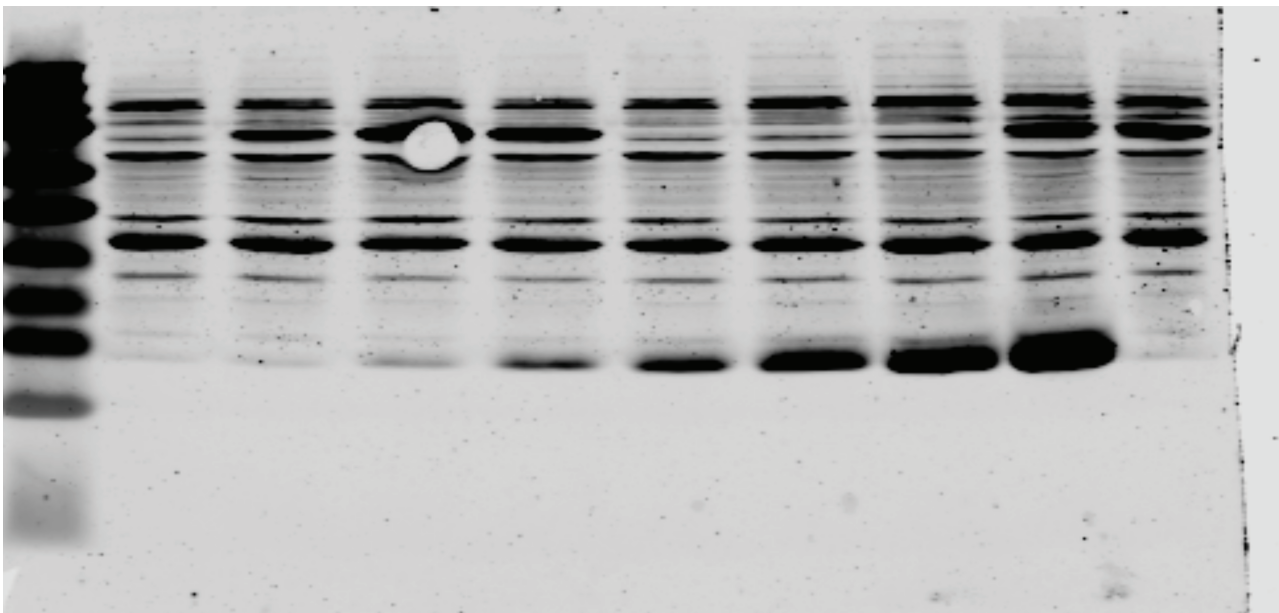

Flag

Supplement: Figure 3—source data 3. [file elife-103705-fig3-data3.zip › Figure 3-source data 2- PDF files containing originall western blots for Figure 3a,indicating the relevant bands and treatments./Source data Figure 3a uncropped western blots 3.pdf]

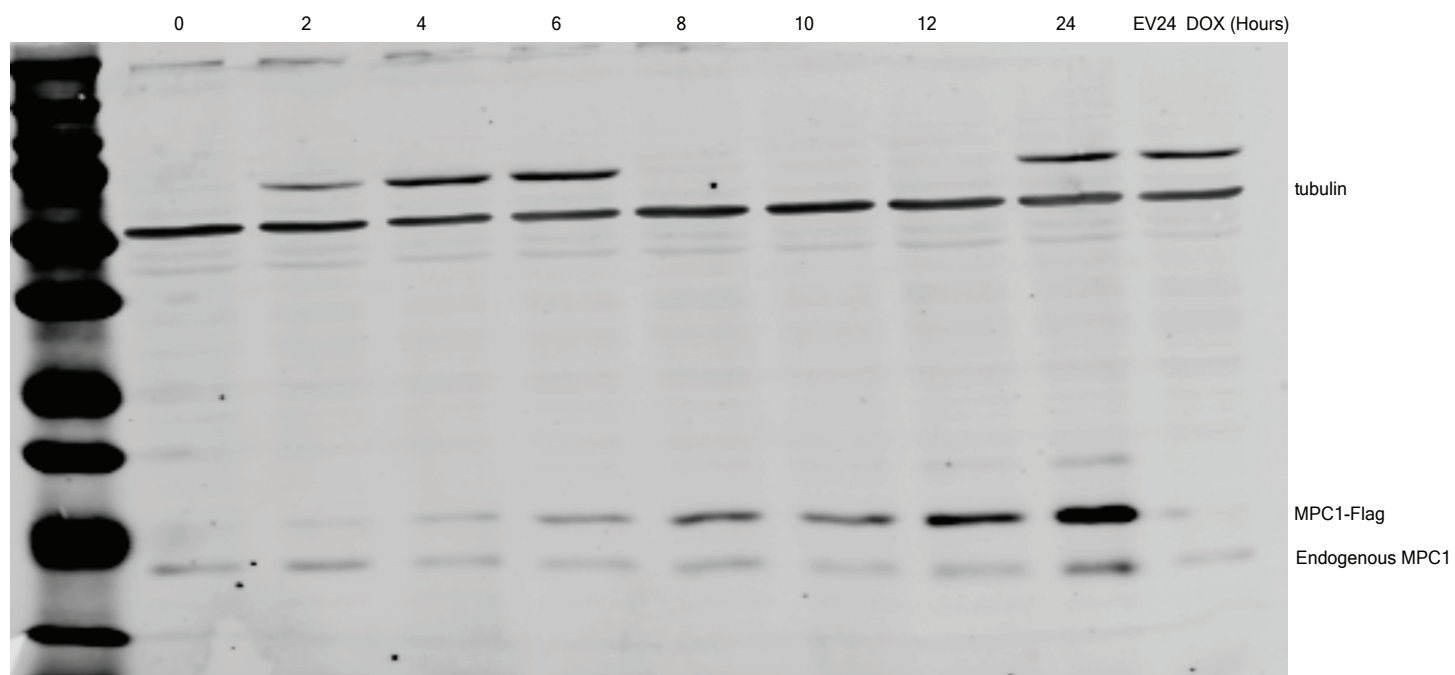

Supplement: Figure 3—source data 3. [file elife-103705-fig3-data3.zip › Figure 3-source data 2- PDF files containing originall western blots for Figure 3a,indicating the relevant bands and treatments./Source data Figure 3a uncropped western blots 1.pdf]

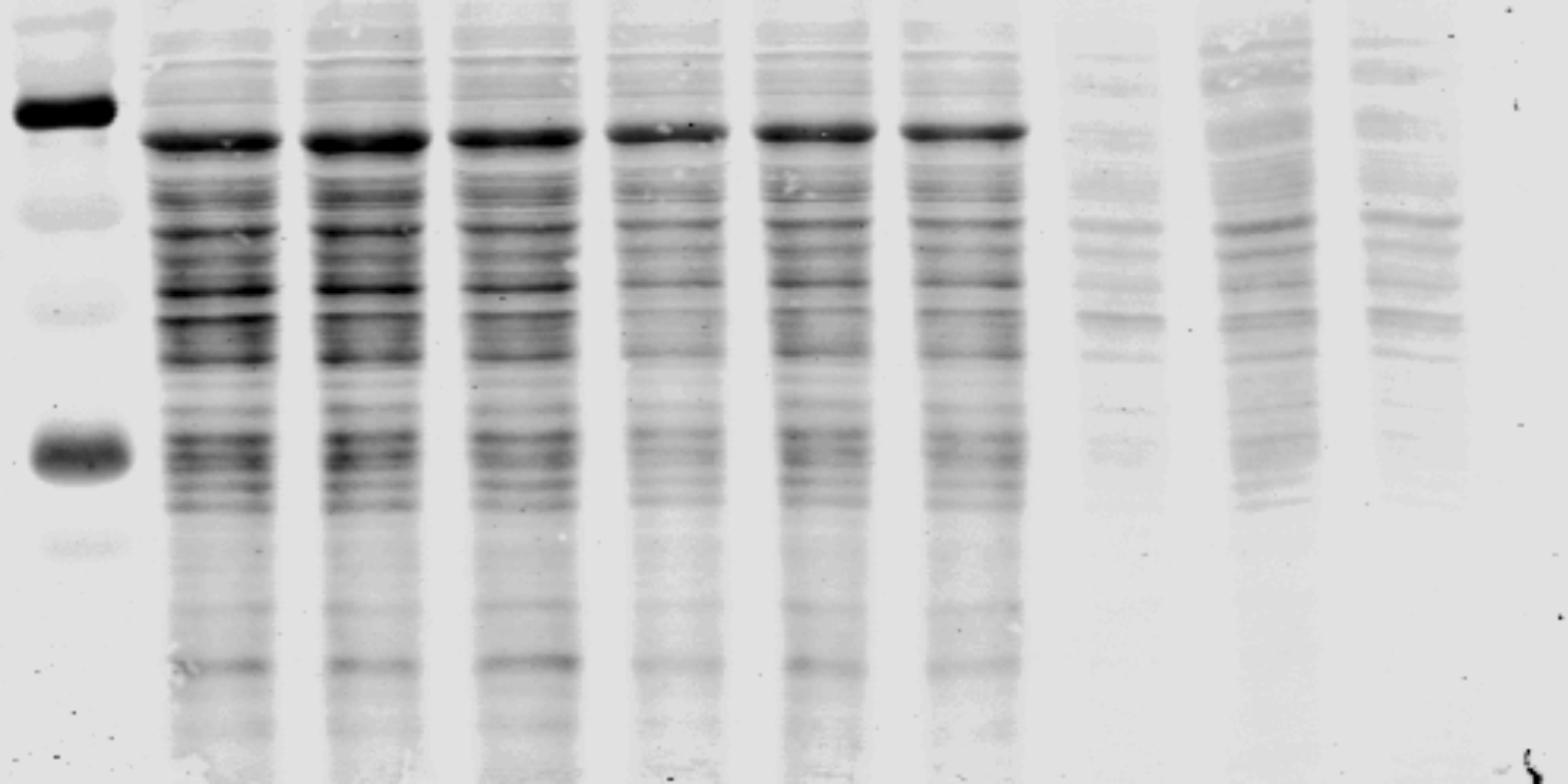

Supplement: Figure 3—source data 4. [file elife-103705-fig3-data4.zip › Figure 3-source data 3-Original files for western blot analysis displayed in Figure 3k/Original files for western blot analysis displayed in Figure 3k 1.tif]

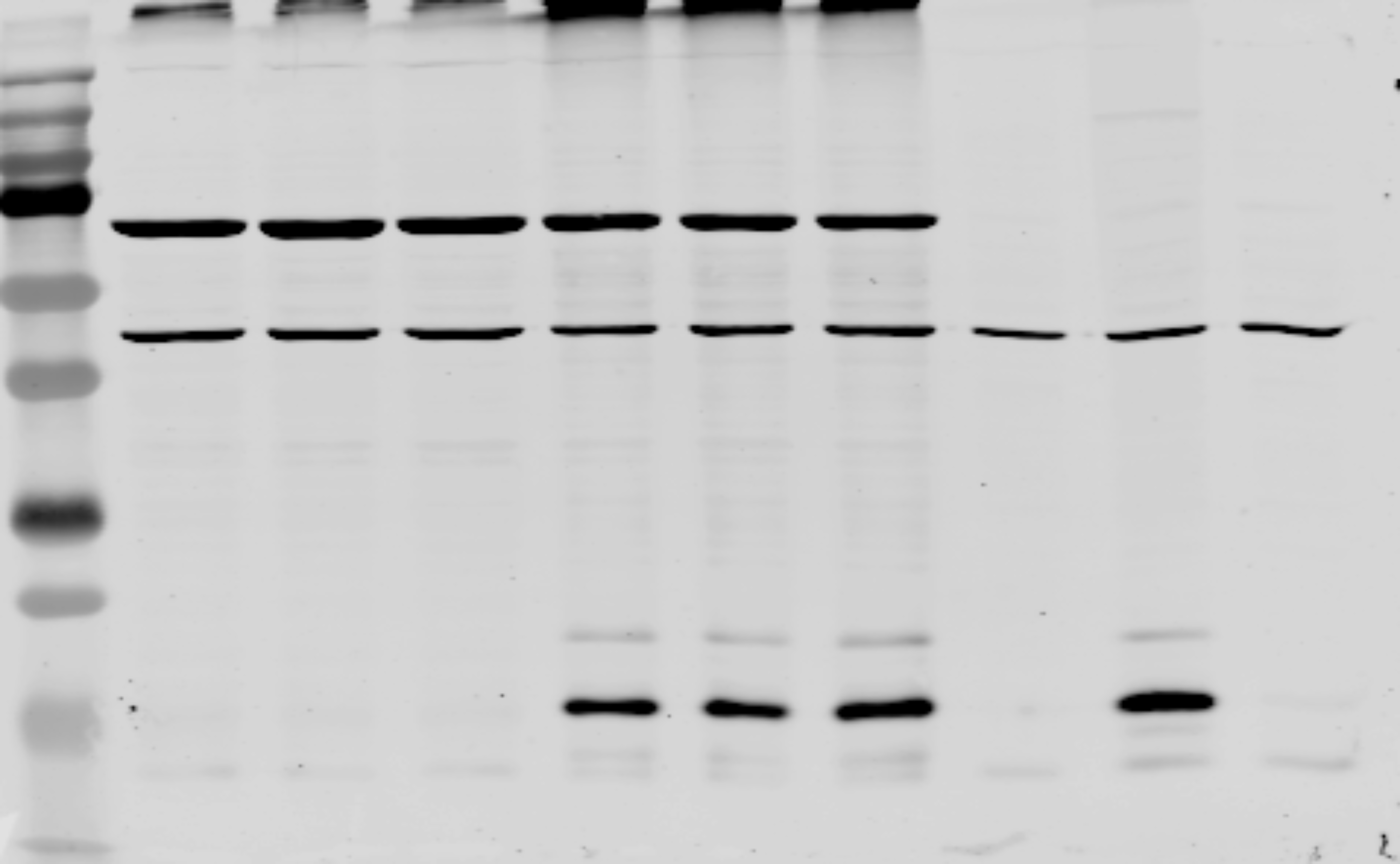

Supplement: Figure 3—source data 4. [file elife-103705-fig3-data4.zip › Figure 3-source data 3-Original files for western blot analysis displayed in Figure 3k/Original files for western blot analysis displayed in Figure 3k 2.tif]

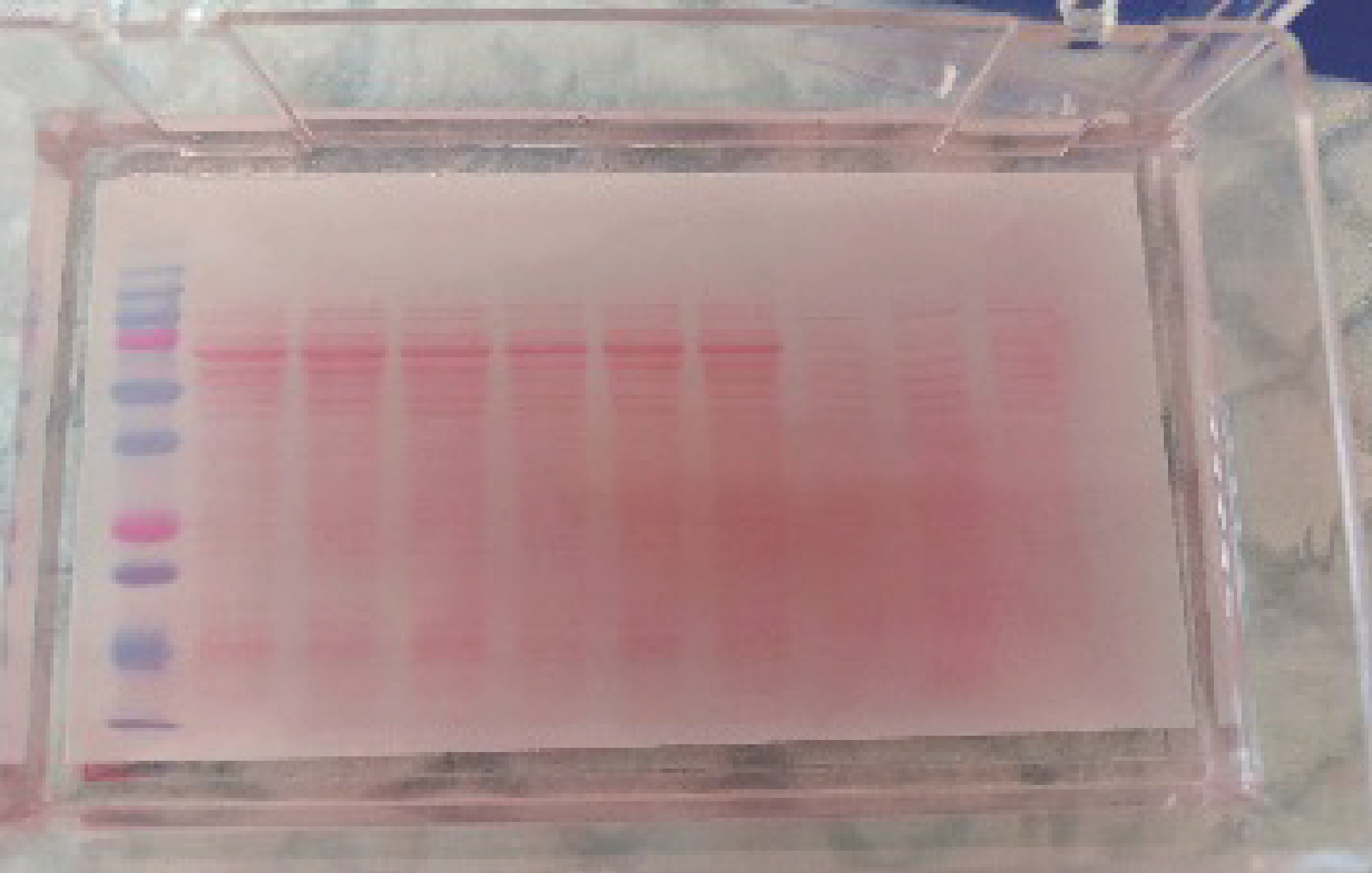

Supplement: Figure 3—source data 5. [file elife-103705-fig3-data5.zip › Figure 3-source data 1- PDF files containing originall western blots for Figure 3k,indicating the relevant bands and treatments./Figure 3-source data 1- PDF files containing originall western blots for Figure 3k,indicating the relevant bands and treatments 3.pdf]

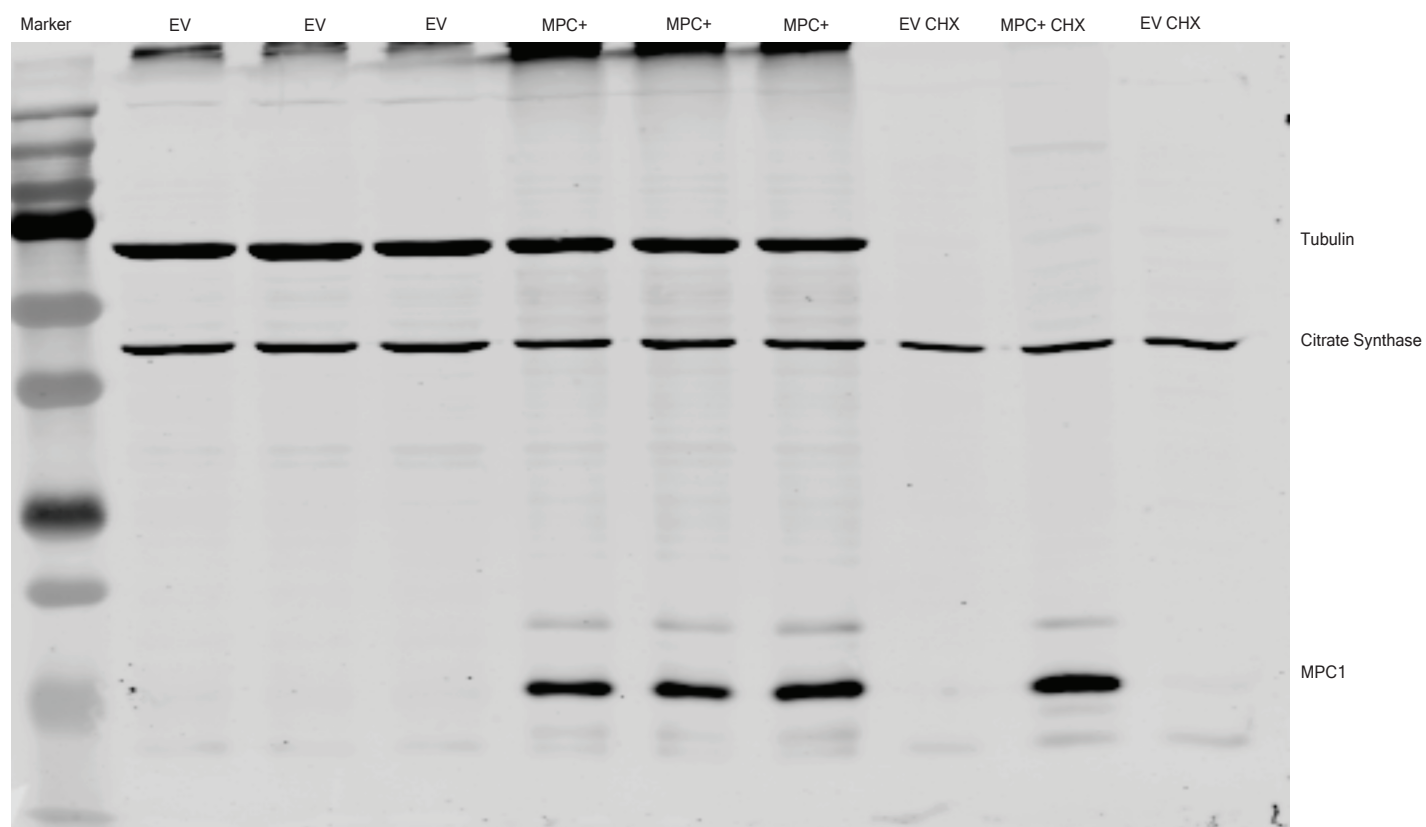

Supplement: Figure 3—source data 5. [file elife-103705-fig3-data5.zip › Figure 3-source data 1- PDF files containing originall western blots for Figure 3k,indicating the relevant bands and treatments./Figure 3-source data 1- PDF files containing originall western blots for Figure 3k,indicating the relevant bands and treatments 2.pdf]

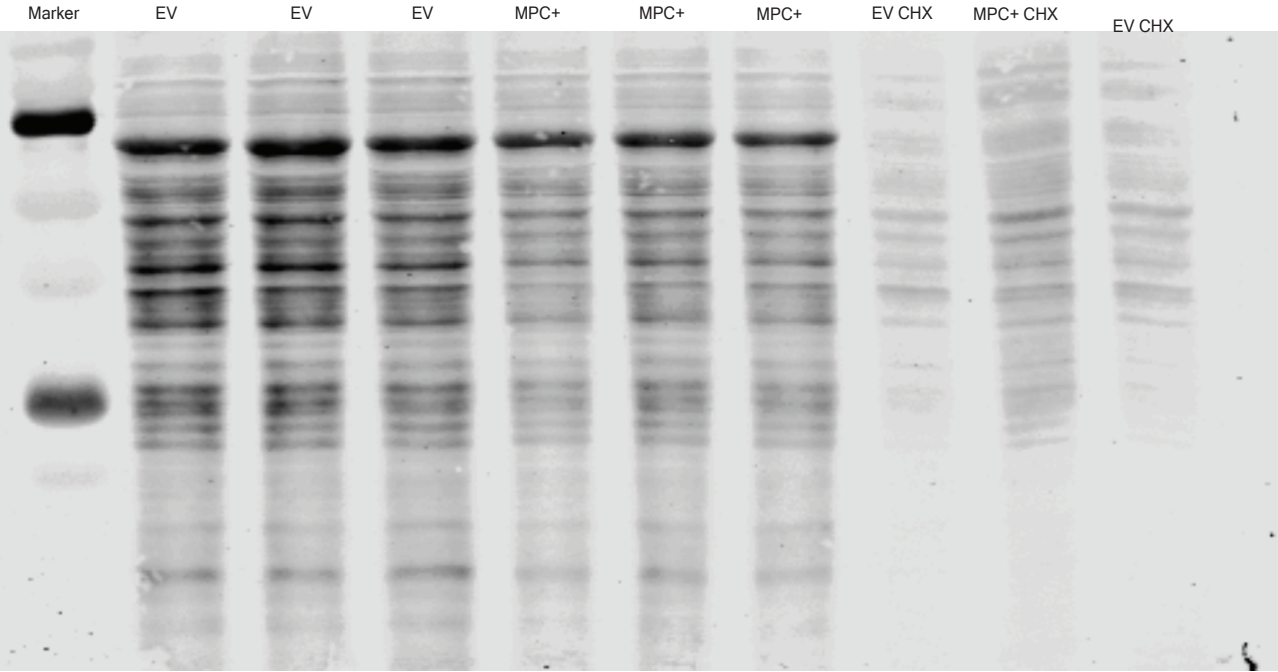

Supplement: Figure 3—source data 5. [file elife-103705-fig3-data5.zip › Figure 3-source data 1- PDF files containing originall western blots for Figure 3k,indicating the relevant bands and treatments./Figure 3-source data 1- PDF files containing originall western blots for Figure 3k,indicating the relevant bands and treatments 1.pdf]

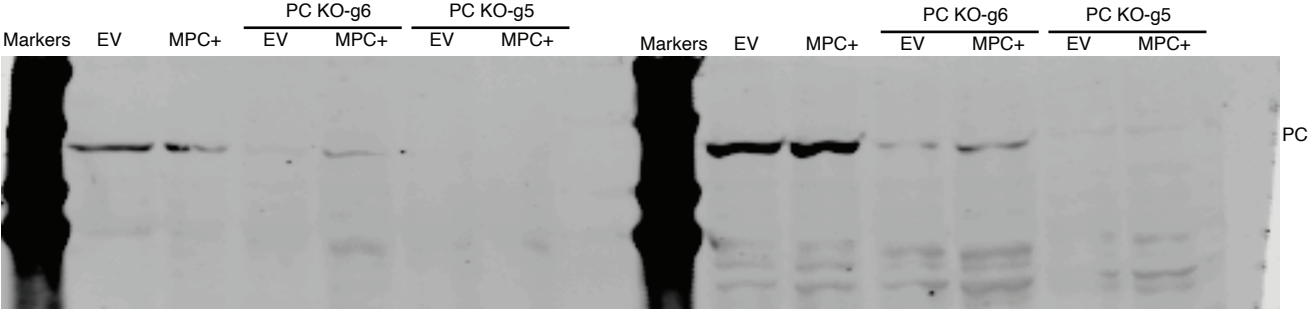

Supplement: Figure 4—figure supplement 2—source data 2. [file elife-103705-fig4-figsupp2-data2.zip › Figure 4-figure supplement 2- source data 1-PDF files containing originall western blots for Figure 4-figure supplement 2e, indicating the relevant bands and treatments./Figure 4-figure supplement 2- PDF files containing originall western blots for Figure 4-figure supplement 2e, indicating the relev]

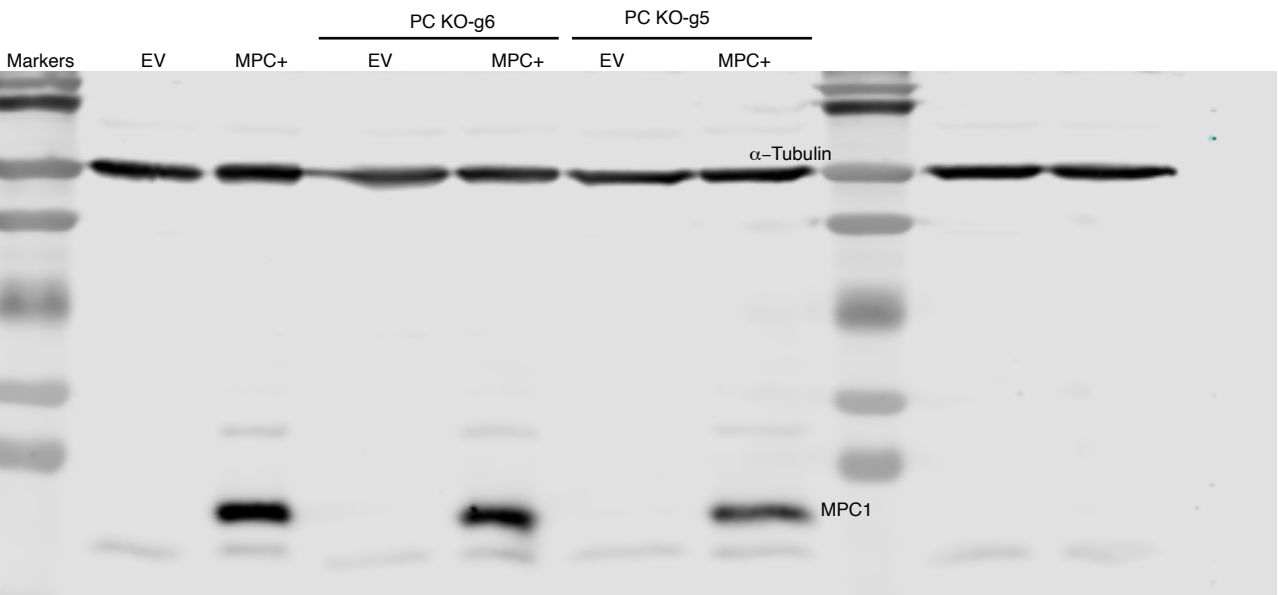

Supplement: Figure 4—figure supplement 2—source data 2. [file elife-103705-fig4-figsupp2-data2.zip › Figure 4-figure supplement 2- source data 1-PDF files containing originall western blots for Figure 4-figure supplement 2e, indicating the relevant bands and treatments./Figure 4-figure supplement - PDF files containing originall western blots for Figure 4-figure supplement 2e, indicating the releva]

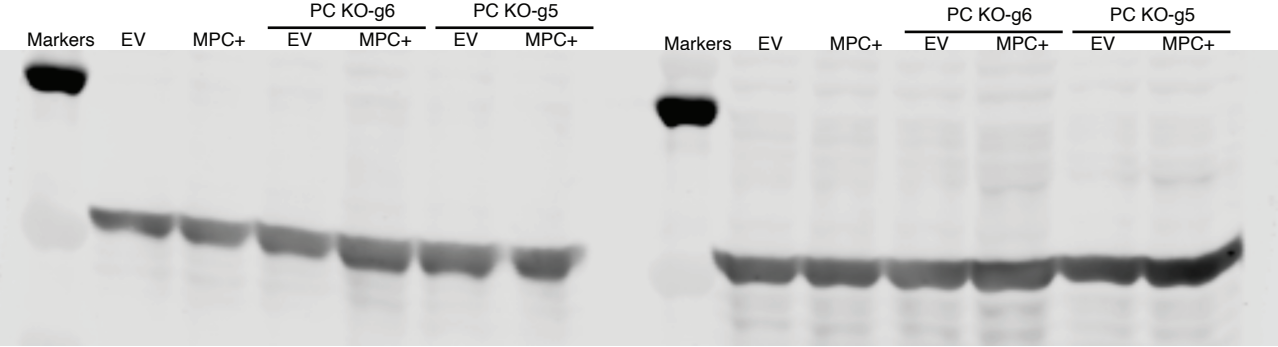

Supplement: Figure 4—figure supplement 2—source data 2. [file elife-103705-fig4-figsupp2-data2.zip › Figure 4-figure supplement 2- source data 1-PDF files containing originall western blots for Figure 4-figure supplement 2e, indicating the relevant bands and treatments./Figure 4-figure supplement 2- PDF files containing originall western blots for Figure 4-figure supplement 2e, indicating the relev]

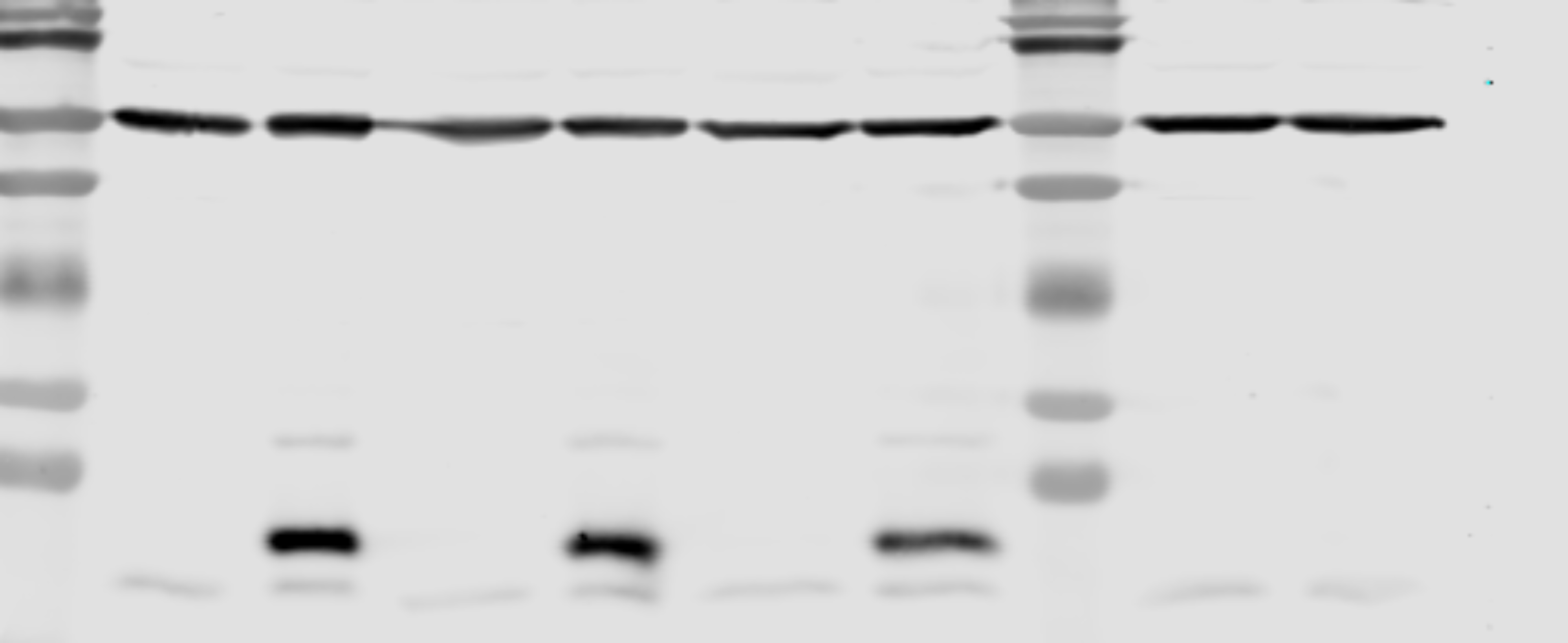

Supplement: Figure 4—figure supplement 2—source data 3. [file elife-103705-fig4-figsupp2-data3.zip › Figure 4-figure supplement 2- source data 3- Original files for western blot analysis displayed in Figure 4-figure supplement 2e/Original files for western blot analysis displayed in Figure 4-figure supplement 2e 3.tif]

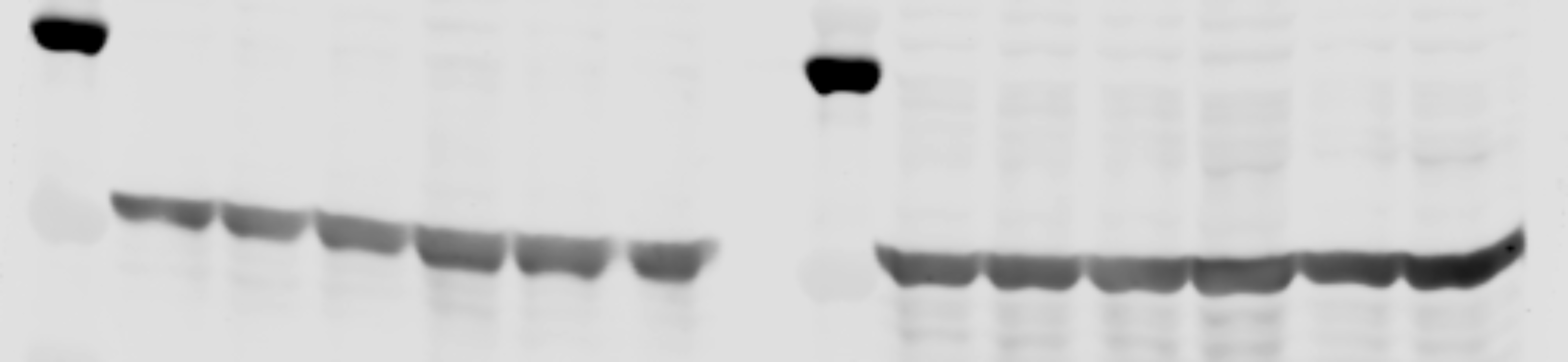

Supplement: Figure 4—figure supplement 2—source data 3. [file elife-103705-fig4-figsupp2-data3.zip › Figure 4-figure supplement 2- source data 3- Original files for western blot analysis displayed in Figure 4-figure supplement 2e/Original files for western blot analysis displayed in Figure 4-figure supplement 2e 2.tif]

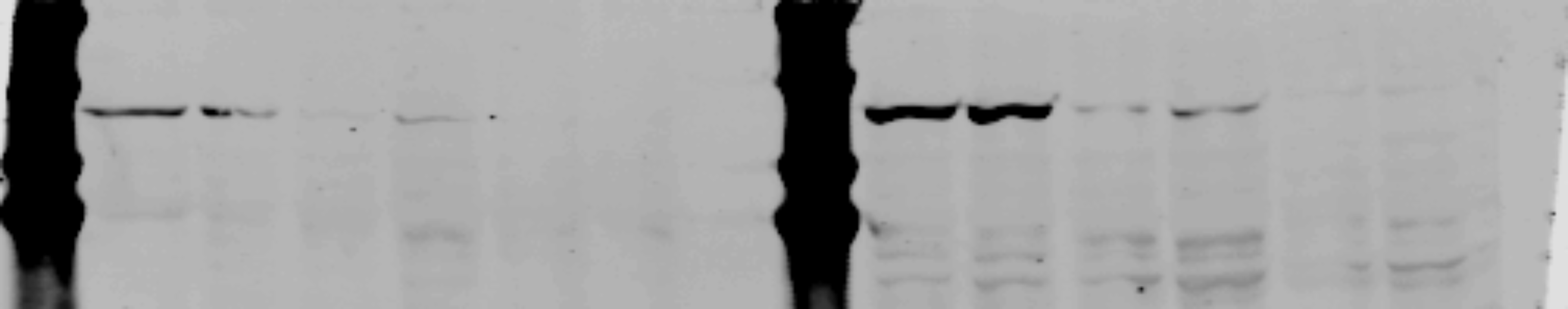

Supplement: Figure 4—figure supplement 2—source data 3. [file elife-103705-fig4-figsupp2-data3.zip › Figure 4-figure supplement 2- source data 3- Original files for western blot analysis displayed in Figure 4-figure supplement 2e/Original files for western blot analysis displayed in Figure 4-figure supplement 2e 1.tif]

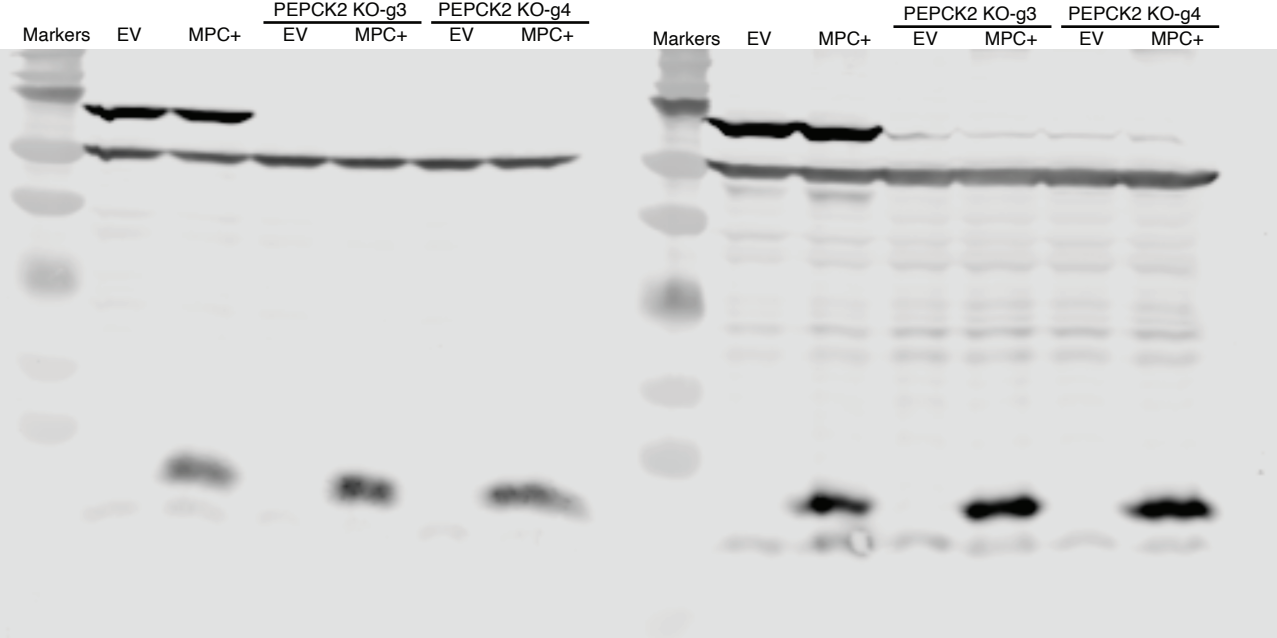

Supplement: Figure 4—figure supplement 2—source data 4. [file elife-103705-fig4-figsupp2-data4.zip › Figure 4-figure supplement 2- source data 2- PDF files containing originall western blots for Figure 4-figure supplement 2m, indicating the relevant bands and treatments./Figure 4-figure supplement 2- PDF files containing originall western blots for Figure 4-figure supplement 2m, indicating the rele]

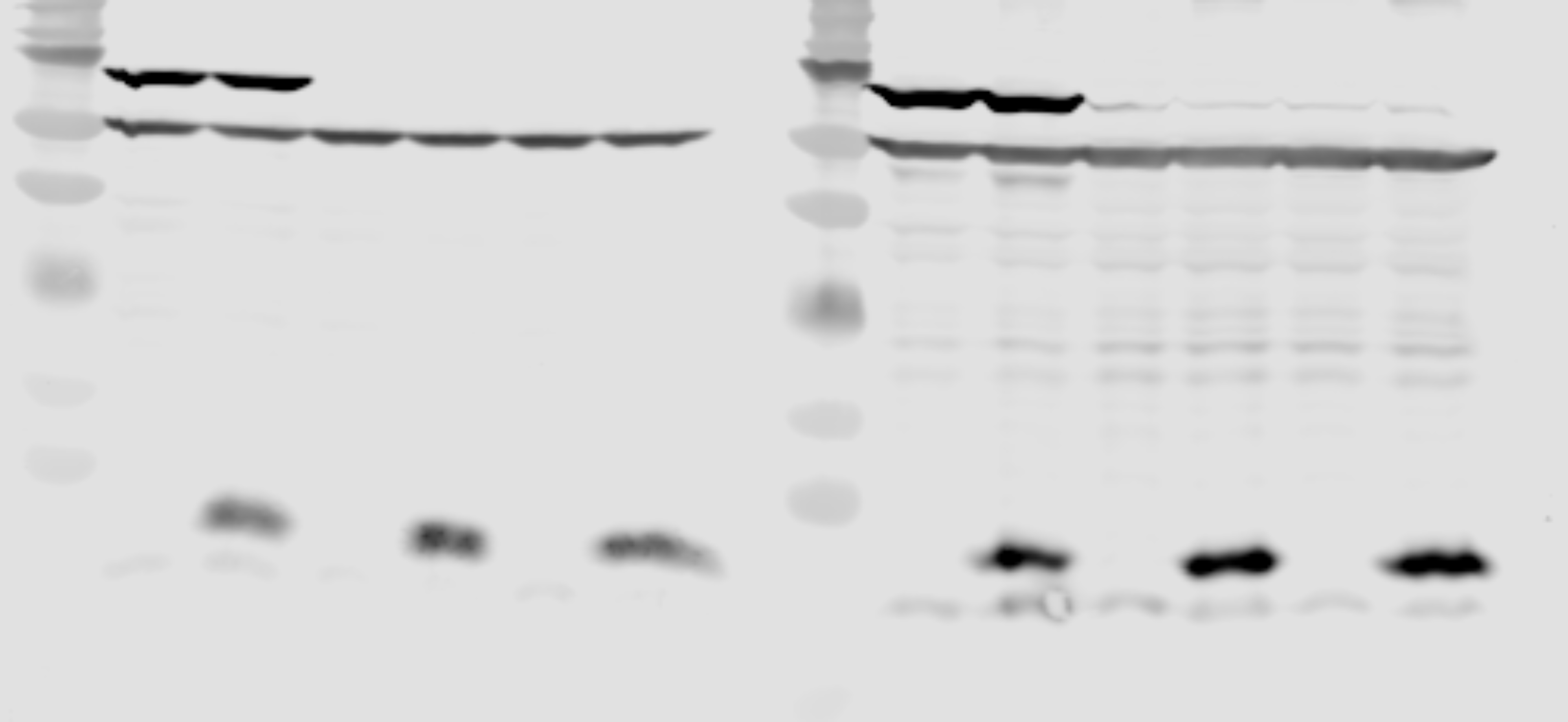

Supplement: Figure 4—figure supplement 2—source data 5. [file elife-103705-fig4-figsupp2-data5.zip › Figure 4-figure supplement 2- source data 4- Original files for western blot analysis displayed in Figure 4-figure supplement 2m/Original files for western blot analysis displayed in Figure 4-figure supplement 2m.tif]

Supplementary Figure 7k

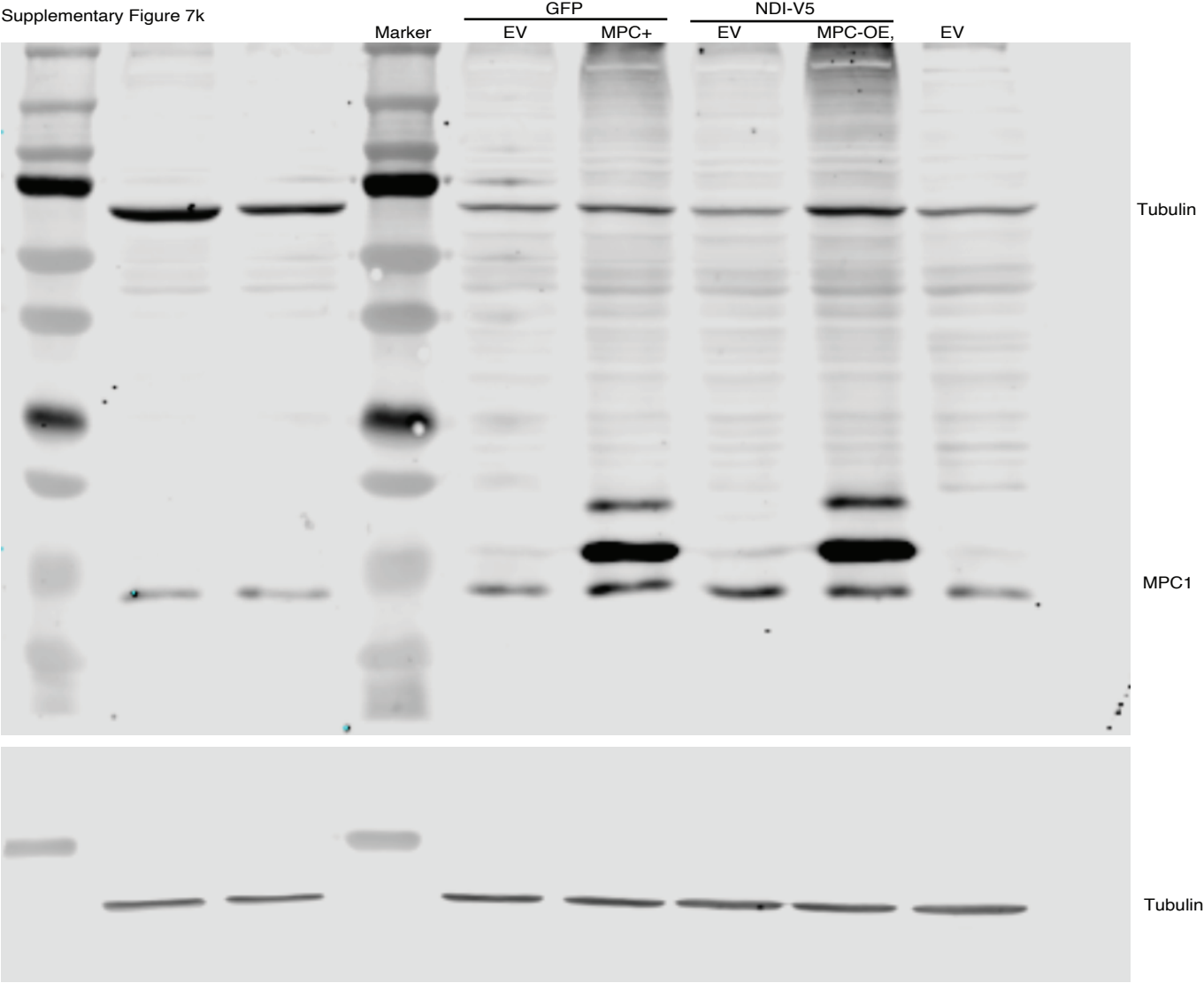

Supplement: Figure 5—figure supplement 1—source data 2. [file elife-103705-fig5-figsupp1-data2.zip › Figure 5- figure supplement 1- source data 1- PDF files containing originall western blots for Figure 5-figure supplement 1k, indicating the relevant bands and treatments./Figure 5-figure supplement 1k- PDF files containing originall western blots for Figure 5-figure supplement 1k, indicating the re]

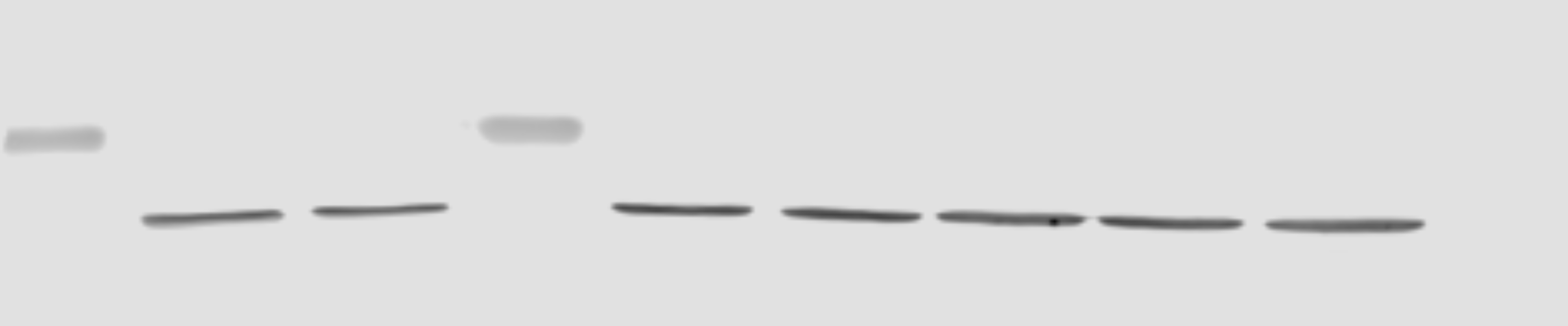

Supplement: Figure 5—figure supplement 1—source data 3. [file elife-103705-fig5-figsupp1-data3.zip › Figure 5- figure supplement 1- source data 3- Original files for western blot analysis displayed in Figure 5-figure supplement 1k/Original files for western blot analysis displayed in Figure 5-figure supplement 1k 2.tif]

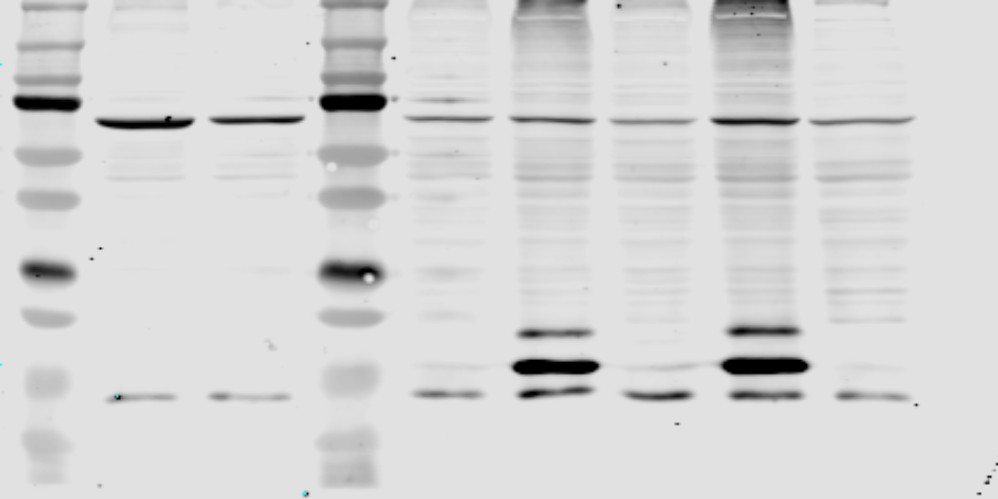

Supplement: Figure 5—figure supplement 1—source data 3. [file elife-103705-fig5-figsupp1-data3.zip › Figure 5- figure supplement 1- source data 3- Original files for western blot analysis displayed in Figure 5-figure supplement 1k/Original files for western blot analysis displayed in Figure 5-figure supplement 1k 1.tif]

0 2 4 6 8 10 12 24 EV24 DOX (Hours)

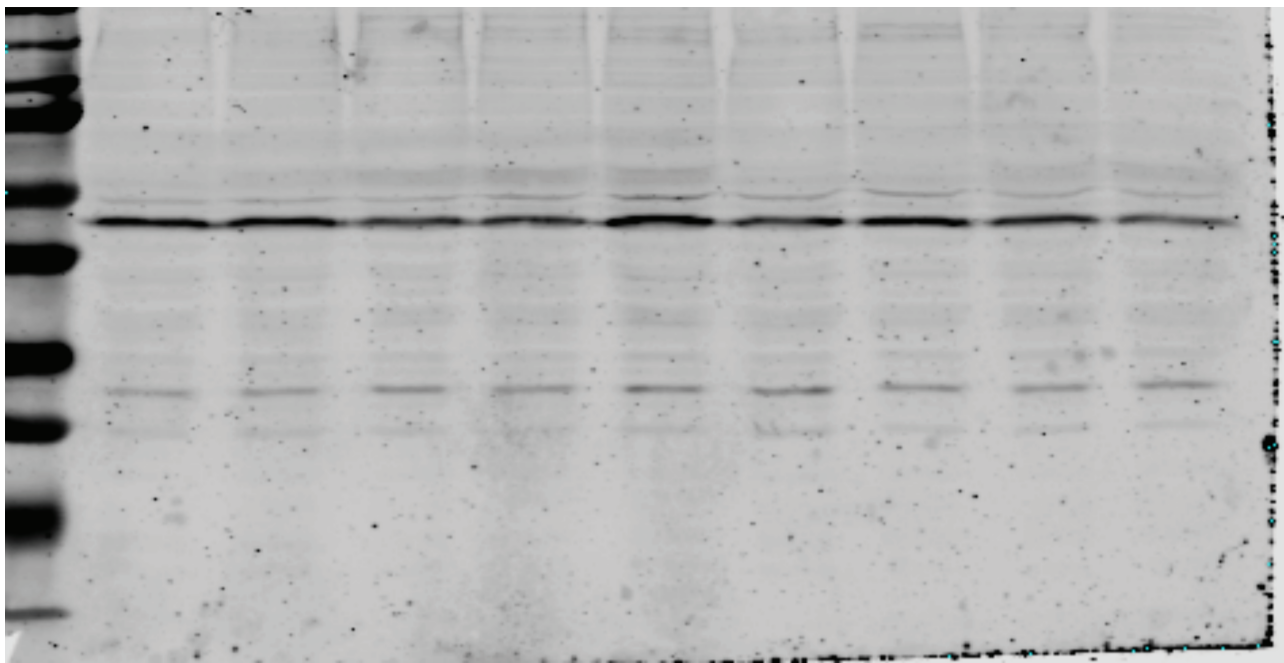

Supplement: Figure 5—figure supplement 1—source data 4. [file elife-103705-fig5-figsupp1-data4.zip › Figure 5-figure supplement 1- source data 2- PDF files containing originall western blots for Figure 5-figure supplement 1b, indicating the relevant bands and treatments./Figure 5-figure supplement 1b - PDF files containing originall western blots for Figure 5-figure supplement 1b, indicating the re]

0

2

4

6

8

10

12

24

EV24 DOX (Hours)

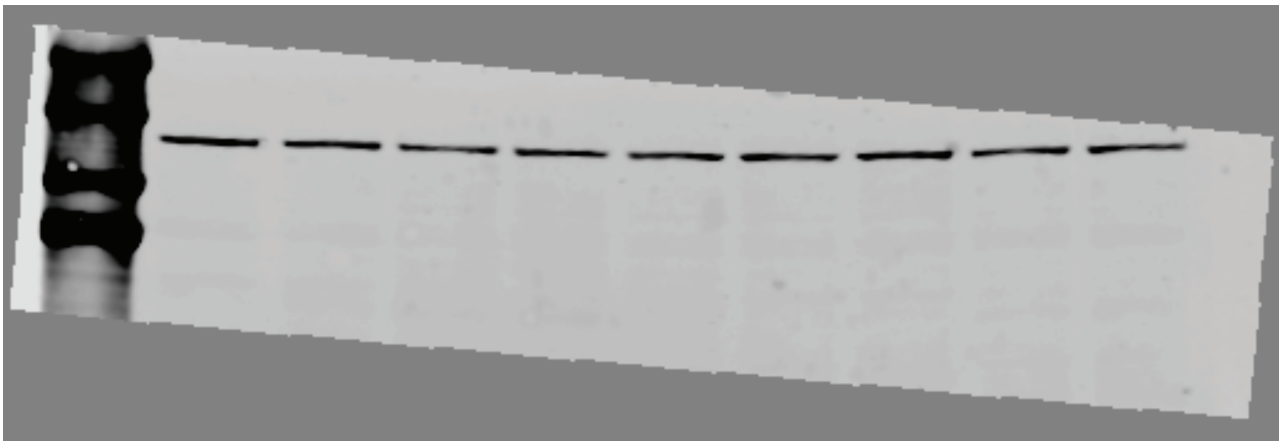

PC

Supplement: Figure 5—figure supplement 1—source data 4. [file elife-103705-fig5-figsupp1-data4.zip › Figure 5-figure supplement 1- source data 2- PDF files containing originall western blots for Figure 5-figure supplement 1b, indicating the relevant bands and treatments./Figure 5-figure supplement 1b - PDF files containing originall western blots for Figure 5-figure supplement 1b, indicating the re]

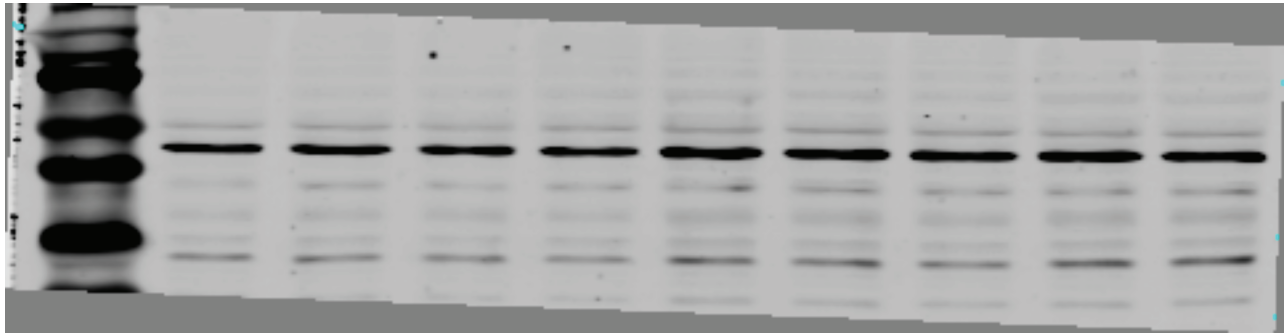

PDH

Supplement: Figure 5—figure supplement 1—source data 4. [file elife-103705-fig5-figsupp1-data4.zip › Figure 5-figure supplement 1- source data 2- PDF files containing originall western blots for Figure 5-figure supplement 1b, indicating the relevant bands and treatments./Figure 5-figure supplement 1b - PDF files containing originall western blots for Figure 5-figure supplement 1b, indicating the re]

0 2 4 6 8 10 12 24 EV24 DOX (Hours)

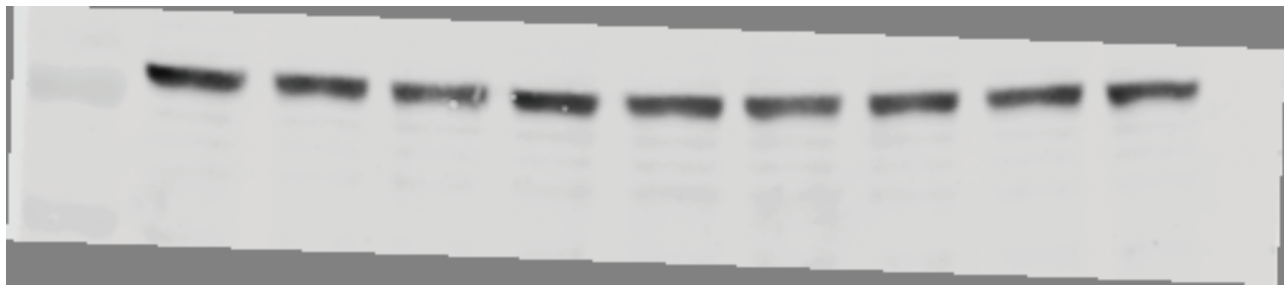

Tubulin

Supplement: Figure 5—figure supplement 1—source data 4. [file elife-103705-fig5-figsupp1-data4.zip › Figure 5-figure supplement 1- source data 2- PDF files containing originall western blots for Figure 5-figure supplement 1b, indicating the relevant bands and treatments./Figure 5-figure supplement 1b - PDF files containing originall western blots for Figure 5-figure supplement 1b, indicating the re]

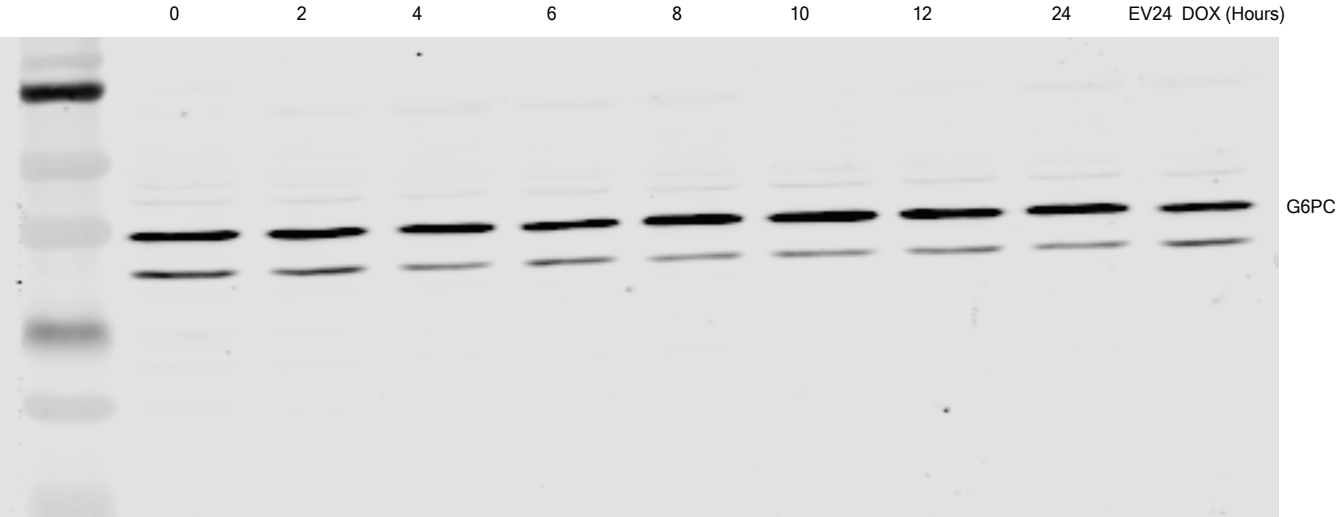

Supplement: Figure 5—figure supplement 1—source data 4. [file elife-103705-fig5-figsupp1-data4.zip › Figure 5-figure supplement 1- source data 2- PDF files containing originall western blots for Figure 5-figure supplement 1b, indicating the relevant bands and treatments./Figure 5-figure supplement 1b - PDF files containing originall western blots for Figure 5-figure supplement 1b, indicating the re]

0 2 4 6 8 10 12 24 EV24 DOX (Hours)

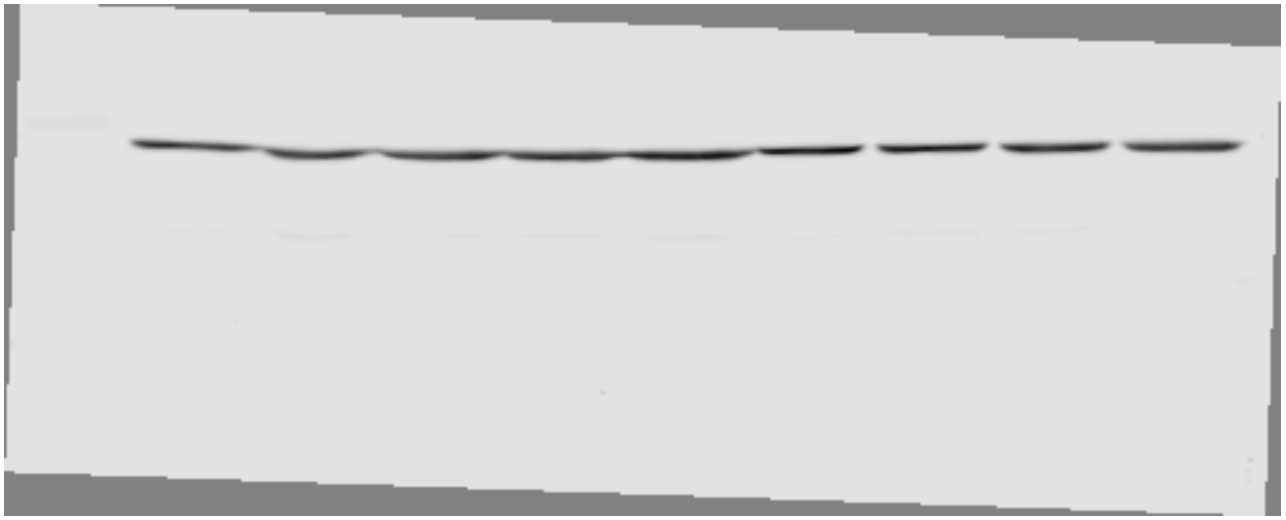

PEPCK2

Supplement: Figure 5—figure supplement 1—source data 4. [file elife-103705-fig5-figsupp1-data4.zip › Figure 5-figure supplement 1- source data 2- PDF files containing originall western blots for Figure 5-figure supplement 1b, indicating the relevant bands and treatments./Figure 5-figure supplement 1b - PDF files containing originall western blots for Figure 5-figure supplement 1b, indicating the re]

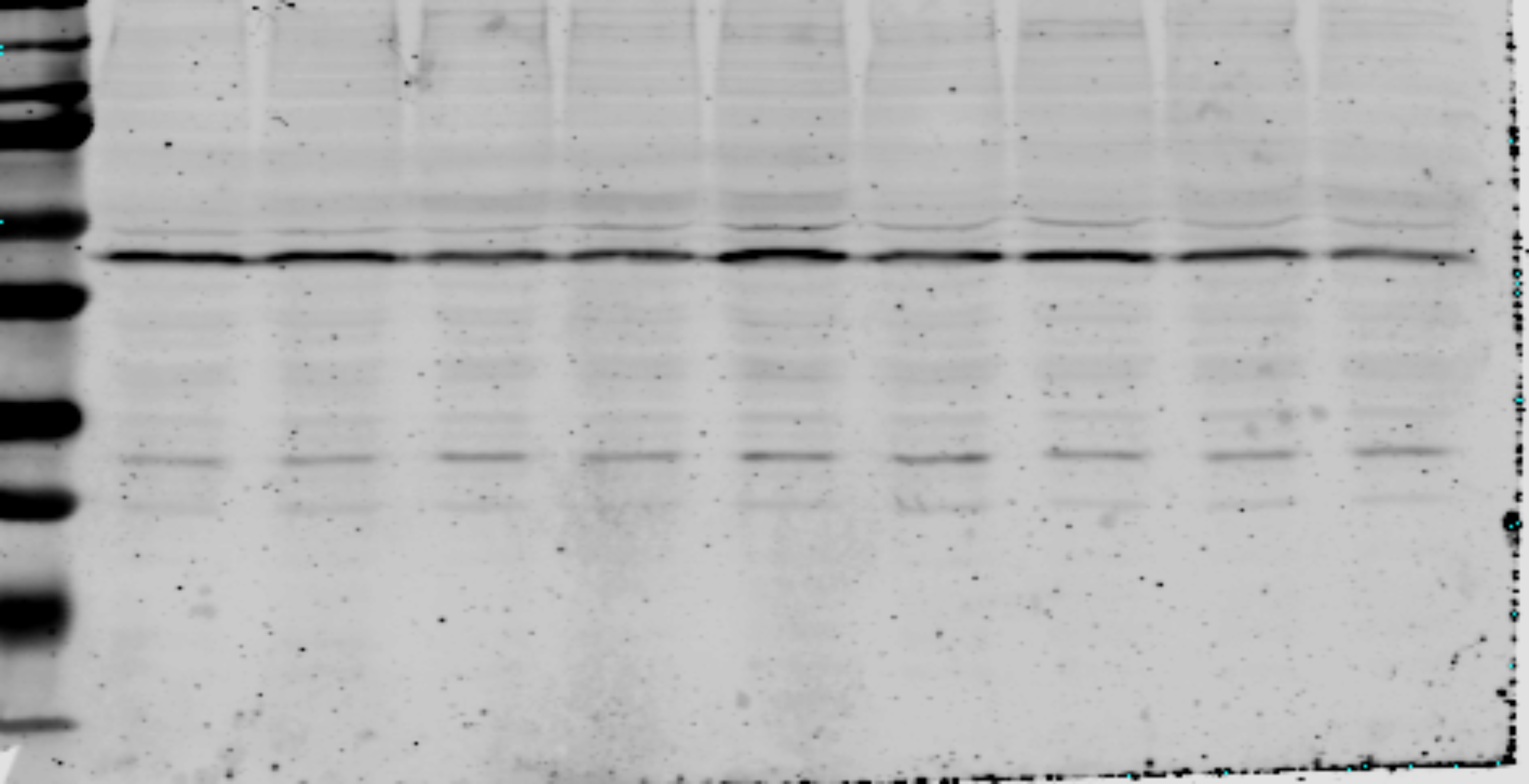

Supplement: Figure 5—figure supplement 1—source data 5. [file elife-103705-fig5-figsupp1-data5.zip › Figure 5- figure supplement 1- source data 4- Original files for western blot analysis displayed in Figure 5-figure supplement 1b/Original files for western blot analysis displayed in Figure 5-figure supplement 1b 1.tif]

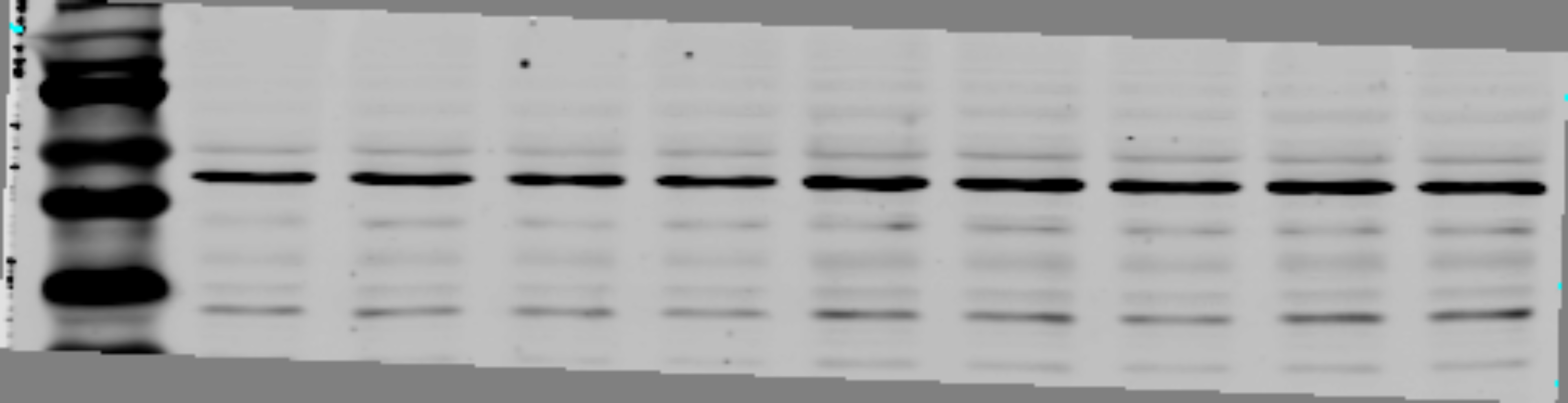

Supplement: Figure 5—figure supplement 1—source data 5. [file elife-103705-fig5-figsupp1-data5.zip › Figure 5- figure supplement 1- source data 4- Original files for western blot analysis displayed in Figure 5-figure supplement 1b/Original files for western blot analysis displayed in Figure 5-figure supplement 1b 2.tif]

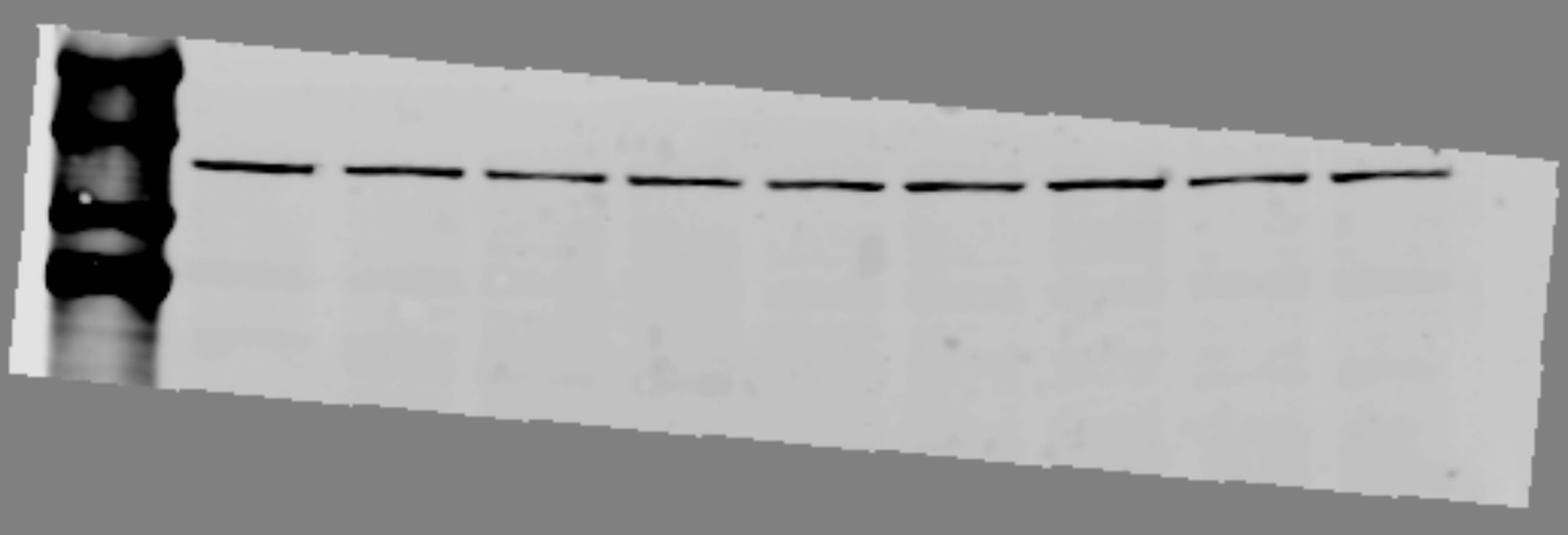

Supplement: Figure 5—figure supplement 1—source data 5. [file elife-103705-fig5-figsupp1-data5.zip › Figure 5- figure supplement 1- source data 4- Original files for western blot analysis displayed in Figure 5-figure supplement 1b/Original files for western blot analysis displayed in Figure 5-figure supplement 1b 3.tif]

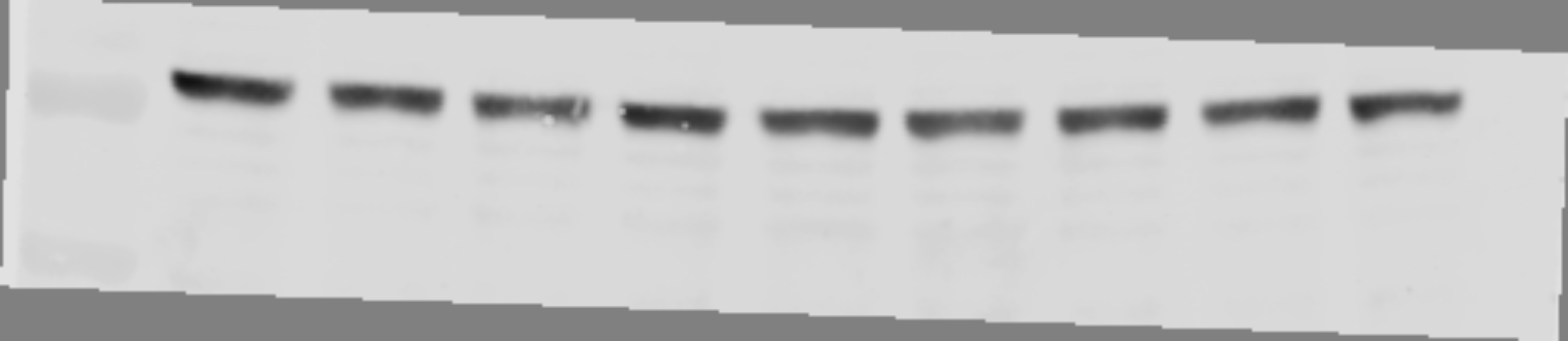

Supplement: Figure 5—figure supplement 1—source data 5. [file elife-103705-fig5-figsupp1-data5.zip › Figure 5- figure supplement 1- source data 4- Original files for western blot analysis displayed in Figure 5-figure supplement 1b/Original files for western blot analysis displayed in Figure 5-figure supplement 1b 6.tif]

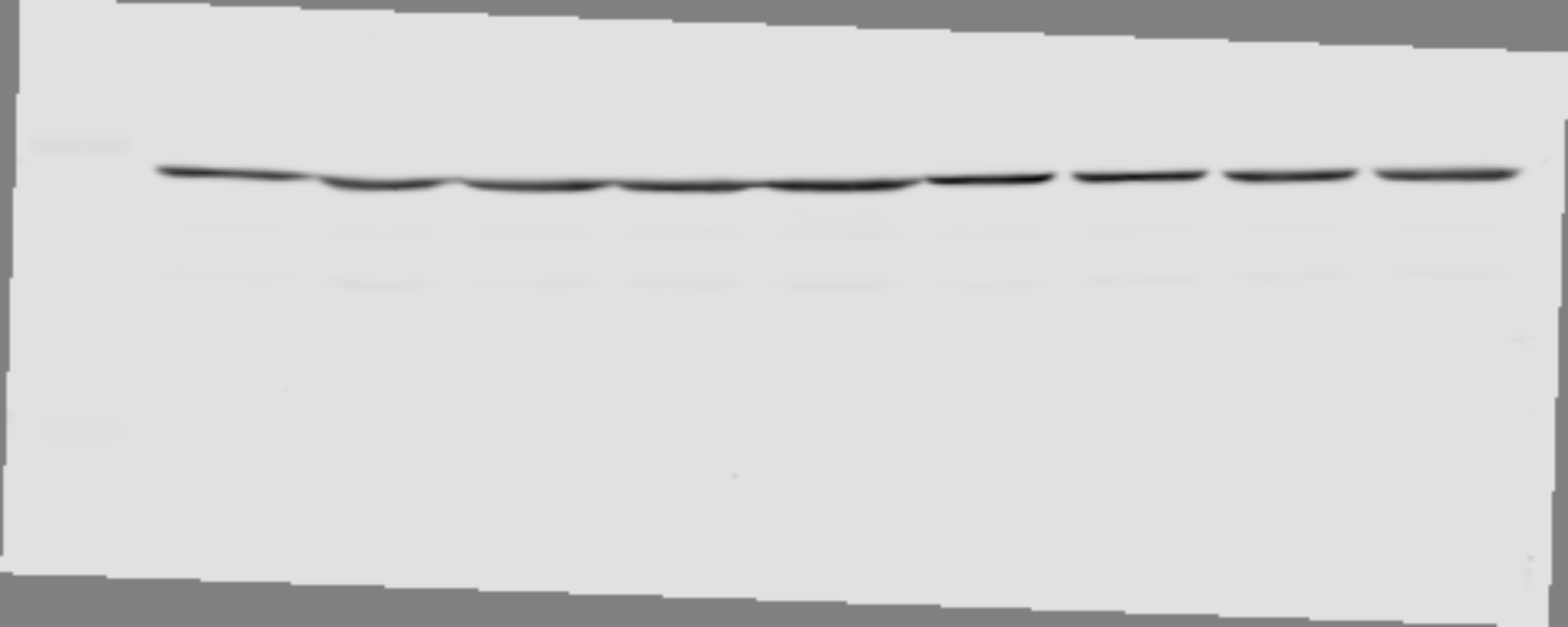

Supplement: Figure 5—figure supplement 1—source data 5. [file elife-103705-fig5-figsupp1-data5.zip › Figure 5- figure supplement 1- source data 4- Original files for western blot analysis displayed in Figure 5-figure supplement 1b/Original files for western blot analysis displayed in Figure 5-figure supplement 1b 4.tif]

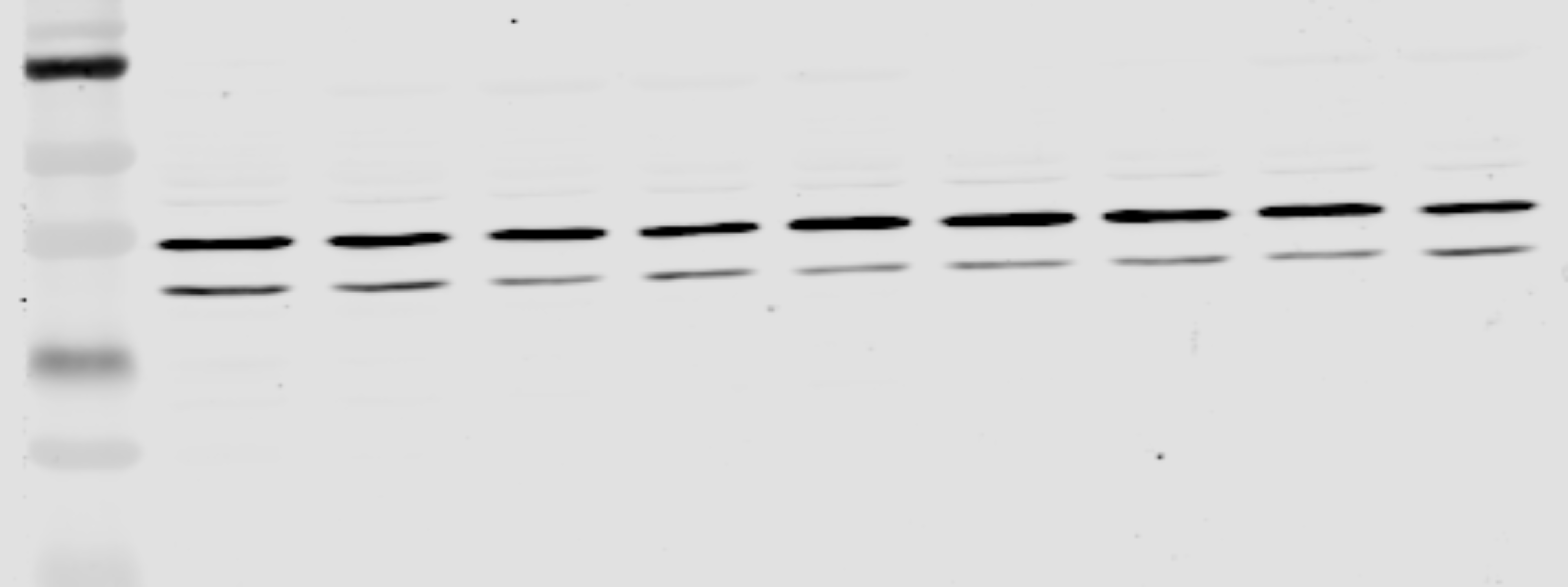

Supplement: Figure 5—figure supplement 1—source data 5. [file elife-103705-fig5-figsupp1-data5.zip › Figure 5- figure supplement 1- source data 4- Original files for western blot analysis displayed in Figure 5-figure supplement 1b/Original files for western blot analysis displayed in Figure 5-figure supplement 1b 5.tif]

| EV   |        |     |     | MPC+ |        |     |     | genotype  |
|------|--------|-----|-----|------|--------|-----|-----|-----------|
| DMSO | UK5099 | NMN | Asp | DMSO | UK5099 | NMN | Asp | Treatment |

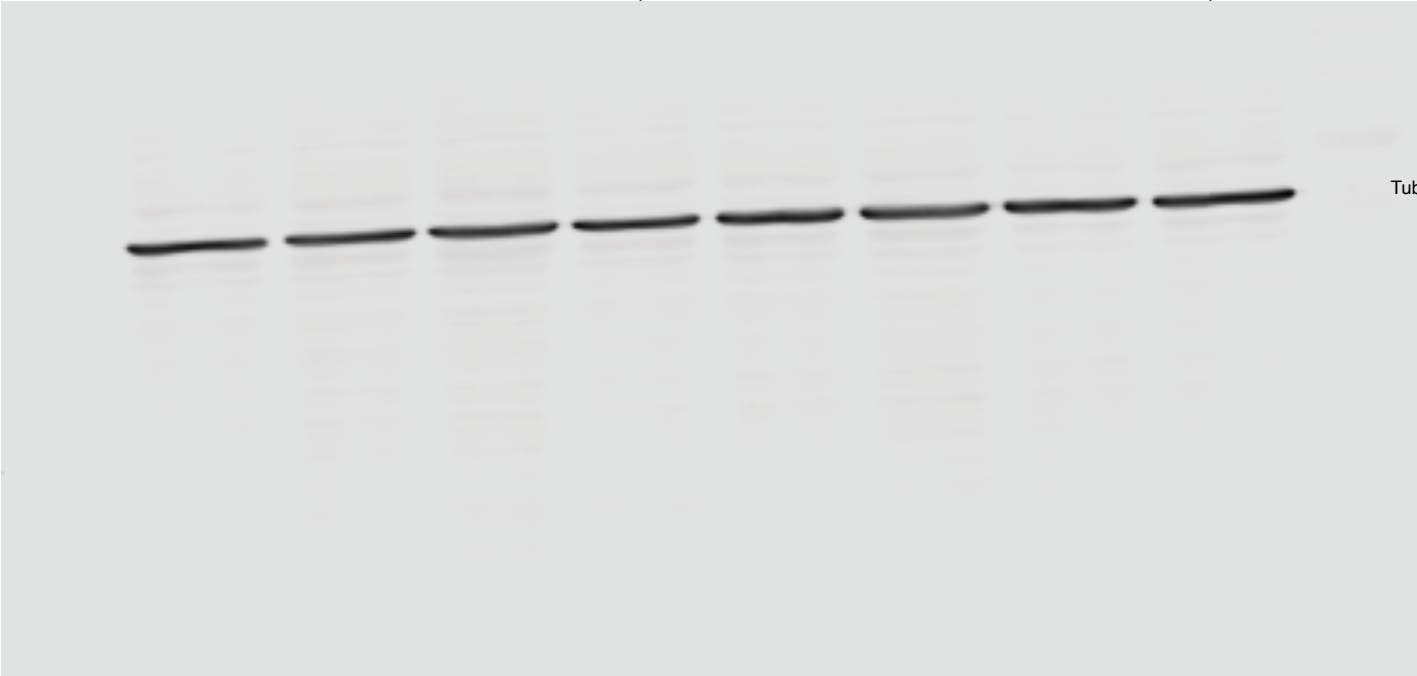

Supplement: Figure 6—source data 2. [file elife-103705-fig6-data2.zip › Figure 6-source data 1- PDF files containing originall western blots for Figure 6i,indicating the relevant bands and treatments./Figure 6-source data 1- PDF files containing originall western blots for Figure 6i,indicating the relevant bands and treatments 4.pdf]

| EV   |        |     |     | MPC+ |        |     |     | genotype  |
|------|--------|-----|-----|------|--------|-----|-----|-----------|
| DMSO | UK5099 | NMN | Asp | DMSO | UK5099 | NMN | Asp | Treatment |

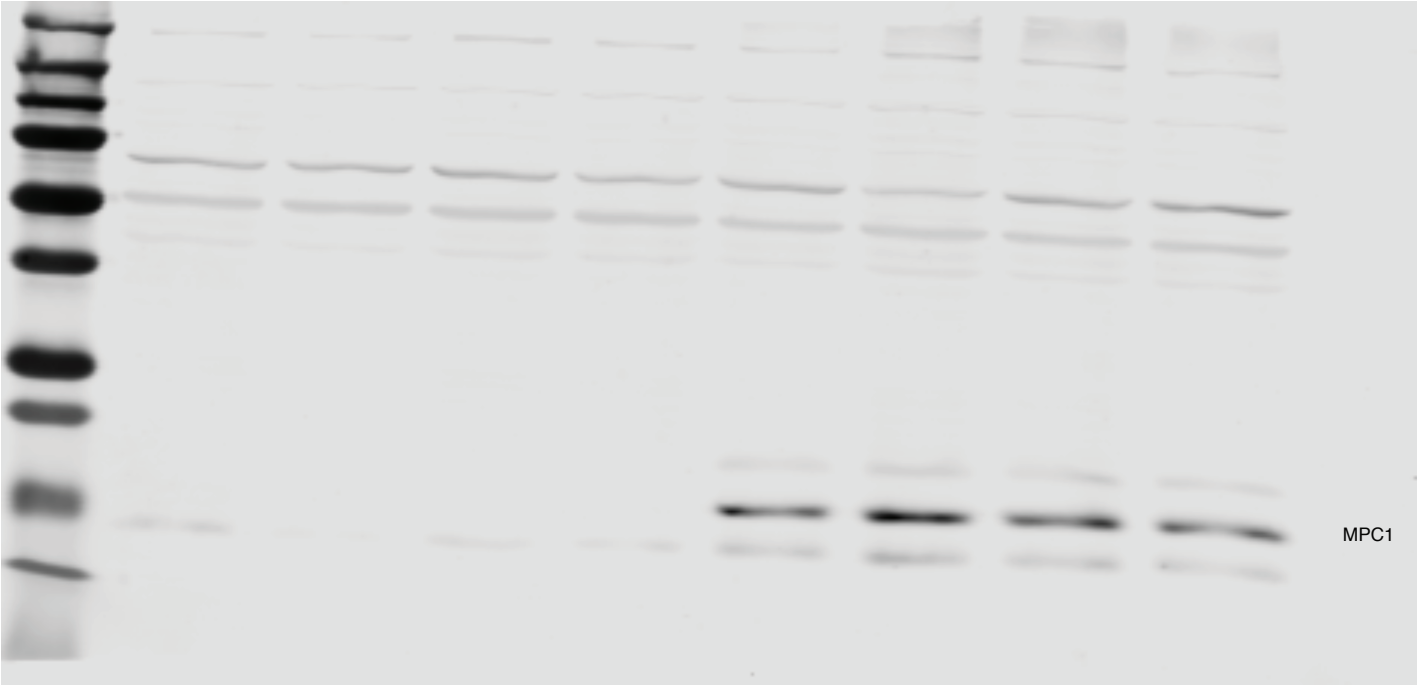

Supplement: Figure 6—source data 2. [file elife-103705-fig6-data2.zip › Figure 6-source data 1- PDF files containing originall western blots for Figure 6i,indicating the relevant bands and treatments./Figure 6-source data 1- PDF files containing originall western blots for Figure 6i,indicating the relevant bands and treatments 5.pdf]

| EV   |        |     |     | MPC+ |        |     |     | genotype  |
|------|--------|-----|-----|------|--------|-----|-----|-----------|
| DMSO | UK5099 | NMN | Asp | DMSO | UK5099 | NMN | Asp | Treatment |

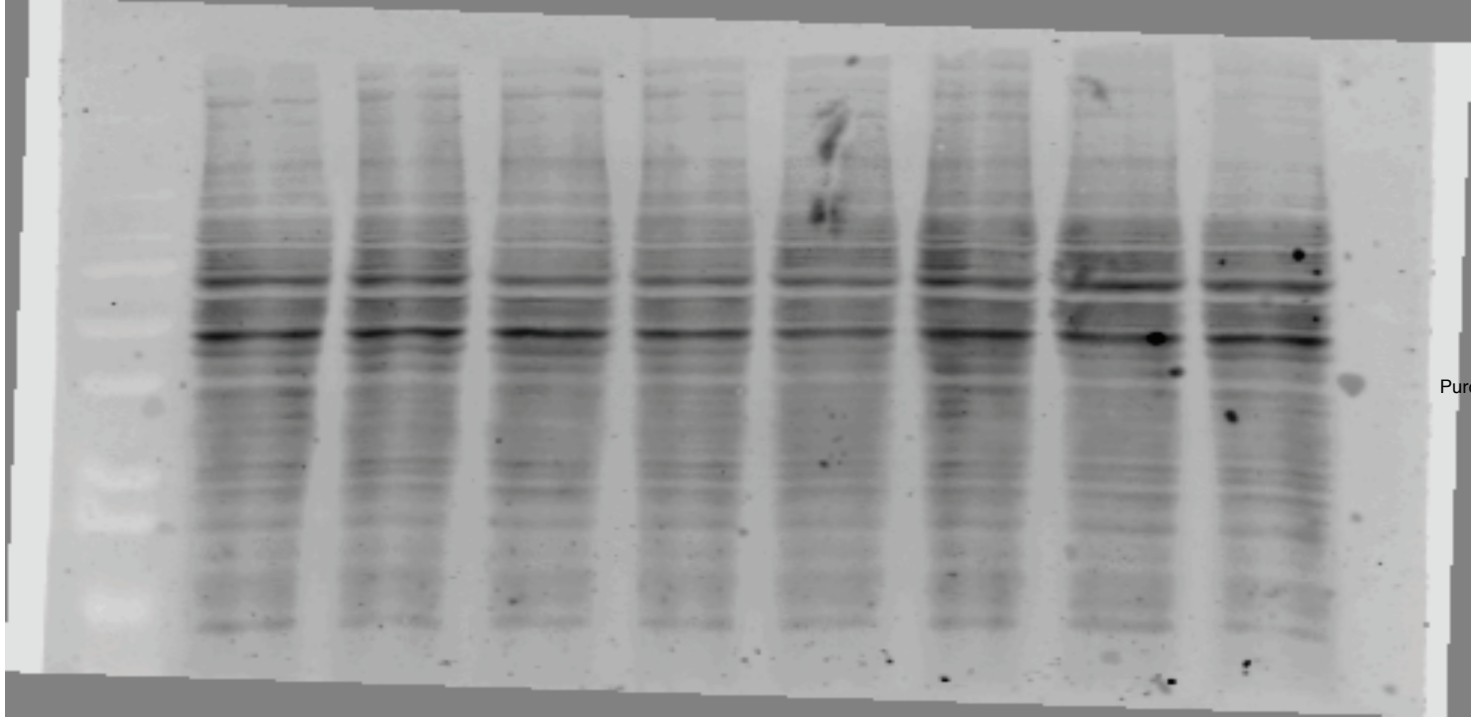

Supplement: Figure 6—source data 2. [file elife-103705-fig6-data2.zip › Figure 6-source data 1- PDF files containing originall western blots for Figure 6i,indicating the relevant bands and treatments./Figure 6-source data 1- PDF files containing originall western blots for Figure 6i,indicating the relevant bands and treatments 2.pdf]

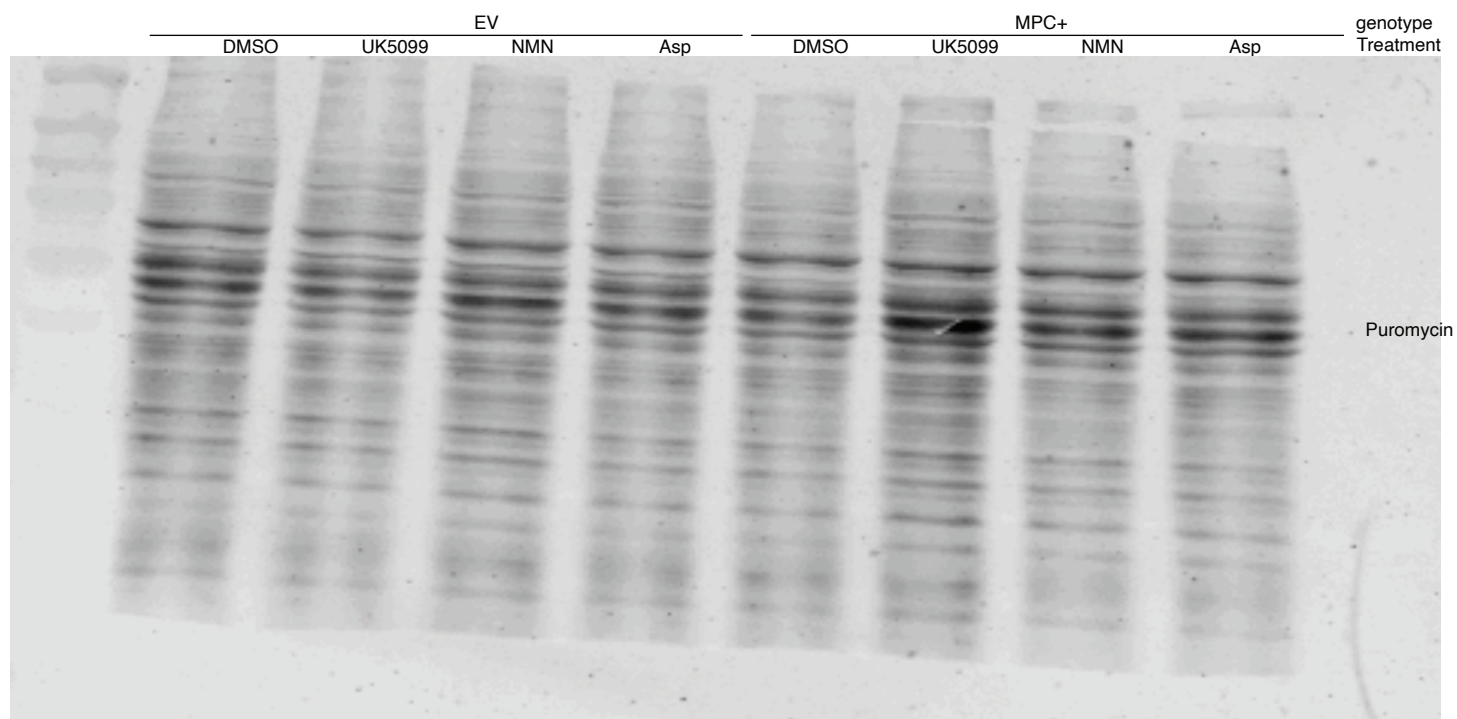

Supplement: Figure 6—source data 2. [file elife-103705-fig6-data2.zip › Figure 6-source data 1- PDF files containing originall western blots for Figure 6i,indicating the relevant bands and treatments./Figure 6-source data 1- PDF files containing originall western blots for Figure 6i,indicating the relevant bands and treatments 3.pdf]

| EV   |        |     |     | MPC+ |        |     |     | genotype  |
|------|--------|-----|-----|------|--------|-----|-----|-----------|
| DMSO | UK5099 | NMN | Asp | DMSO | UK5099 | NMN | Asp | Treatment |

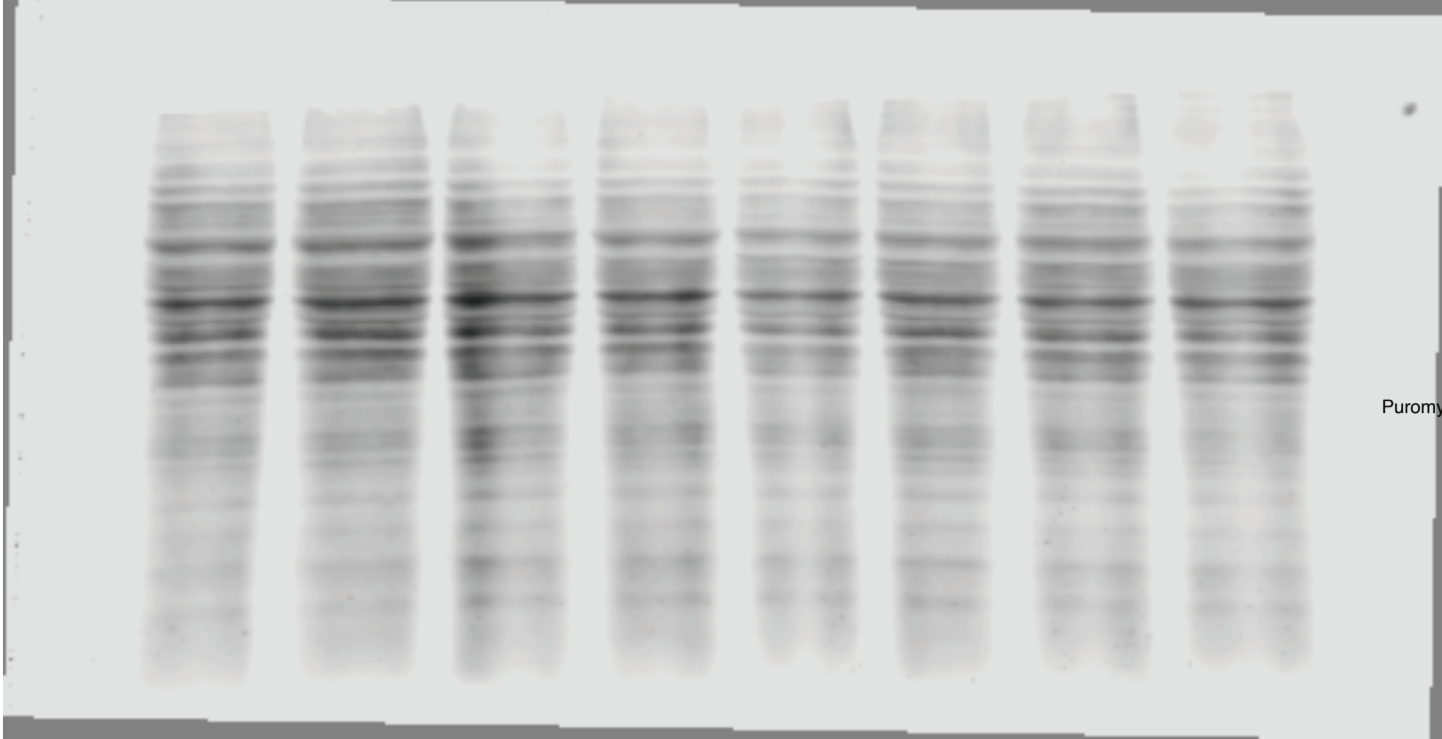

Supplement: Figure 6—source data 2. [file elife-103705-fig6-data2.zip › Figure 6-source data 1- PDF files containing originall western blots for Figure 6i,indicating the relevant bands and treatments./Figure 6-source data 1- PDF files containing originall western blots for Figure 6i,indicating the relevant bands and treatments 1.pdf]

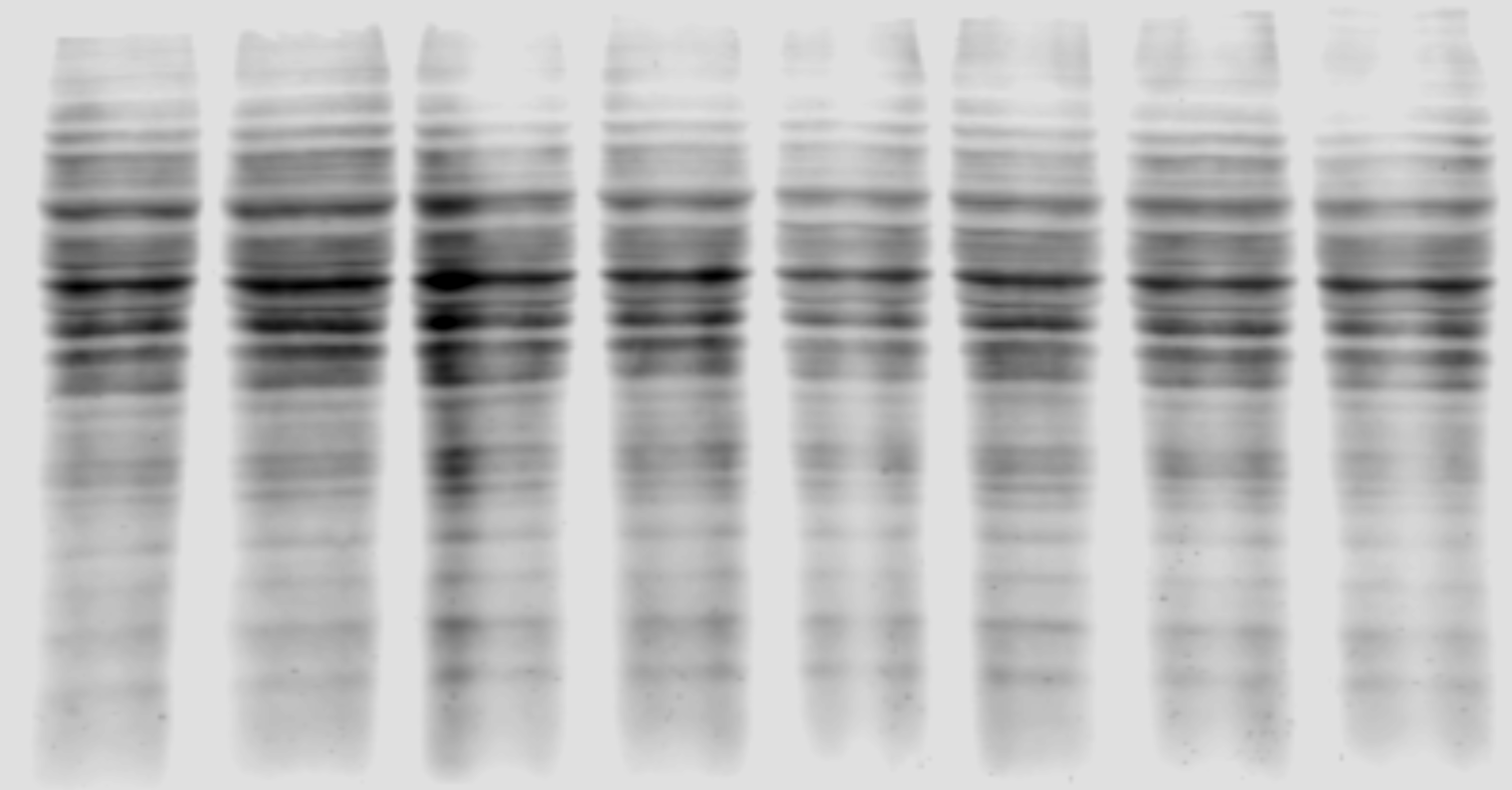

Supplement: Figure 6—source data 3. [file elife-103705-fig6-data3.zip › Figure 6- source data 2-Original files for western blot analysis displayed in Figure 6i/Original files for western blot analysis displayed in Figure 6i 1.tif]

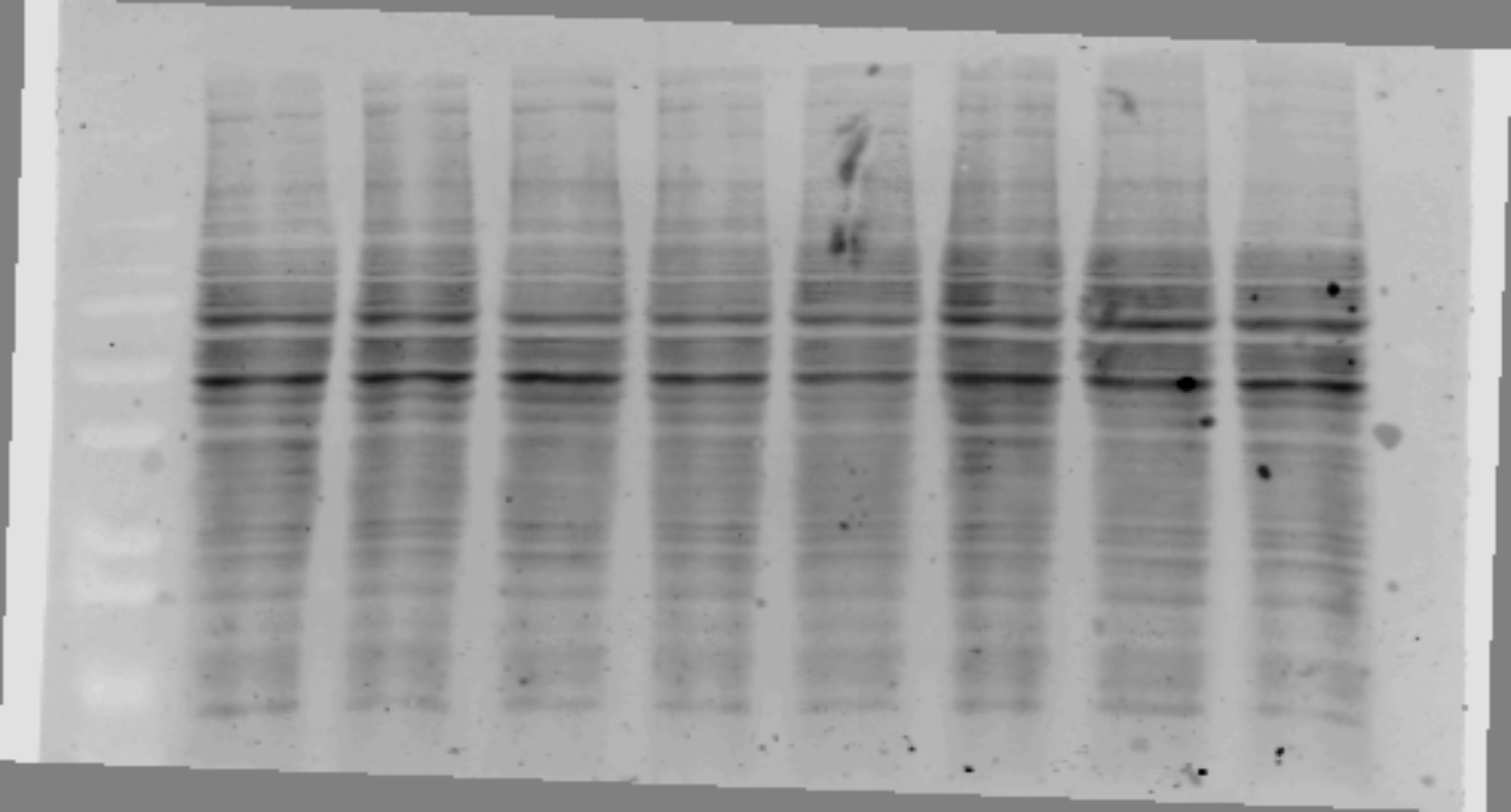

Supplement: Figure 6—source data 3. [file elife-103705-fig6-data3.zip › Figure 6- source data 2-Original files for western blot analysis displayed in Figure 6i/Original files for western blot analysis displayed in Figure 6i 2.tif]

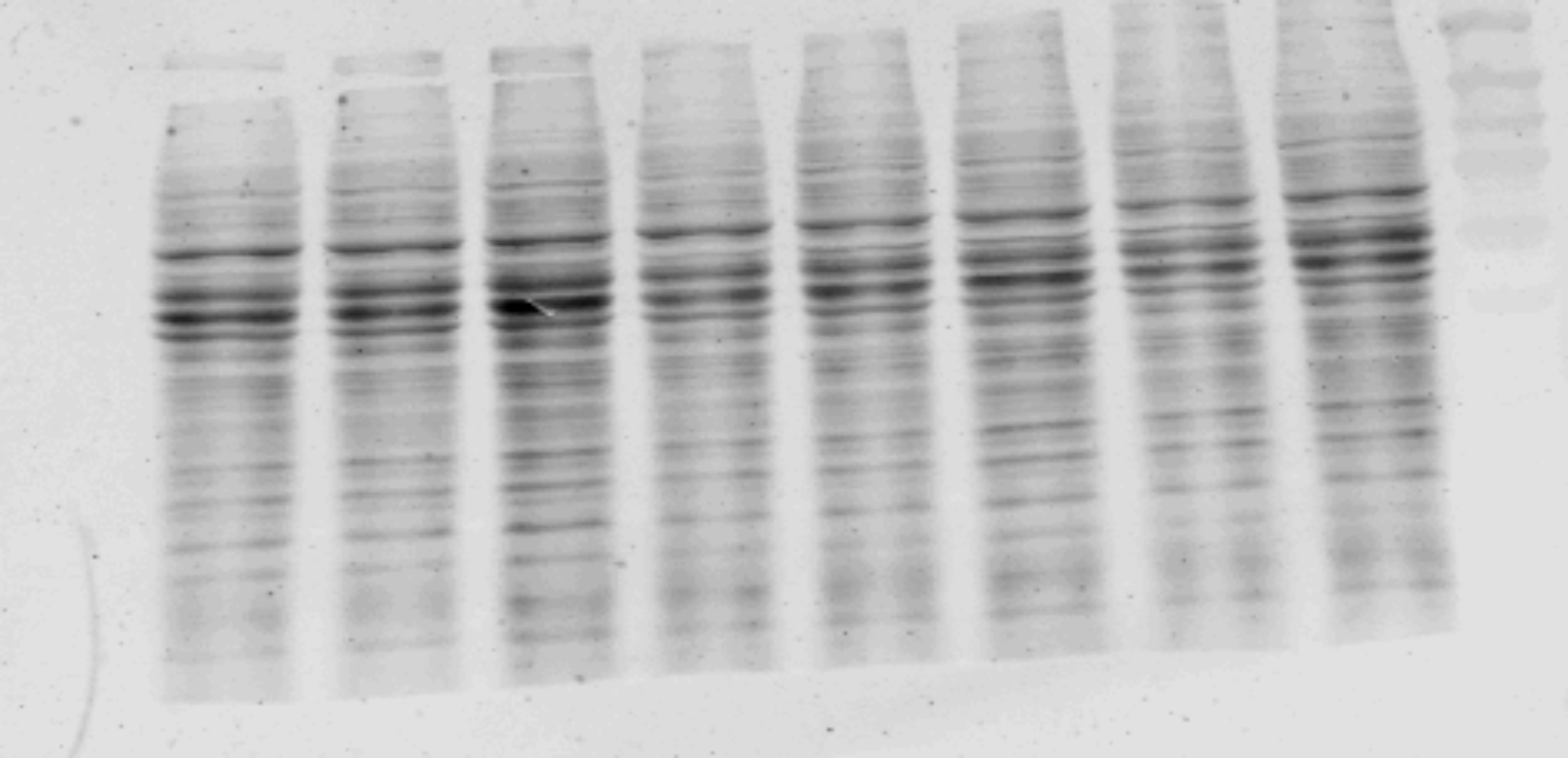

Supplement: Figure 6—source data 3. [file elife-103705-fig6-data3.zip › Figure 6- source data 2-Original files for western blot analysis displayed in Figure 6i/Original files for western blot analysis displayed in Figure 6i 3.tif]

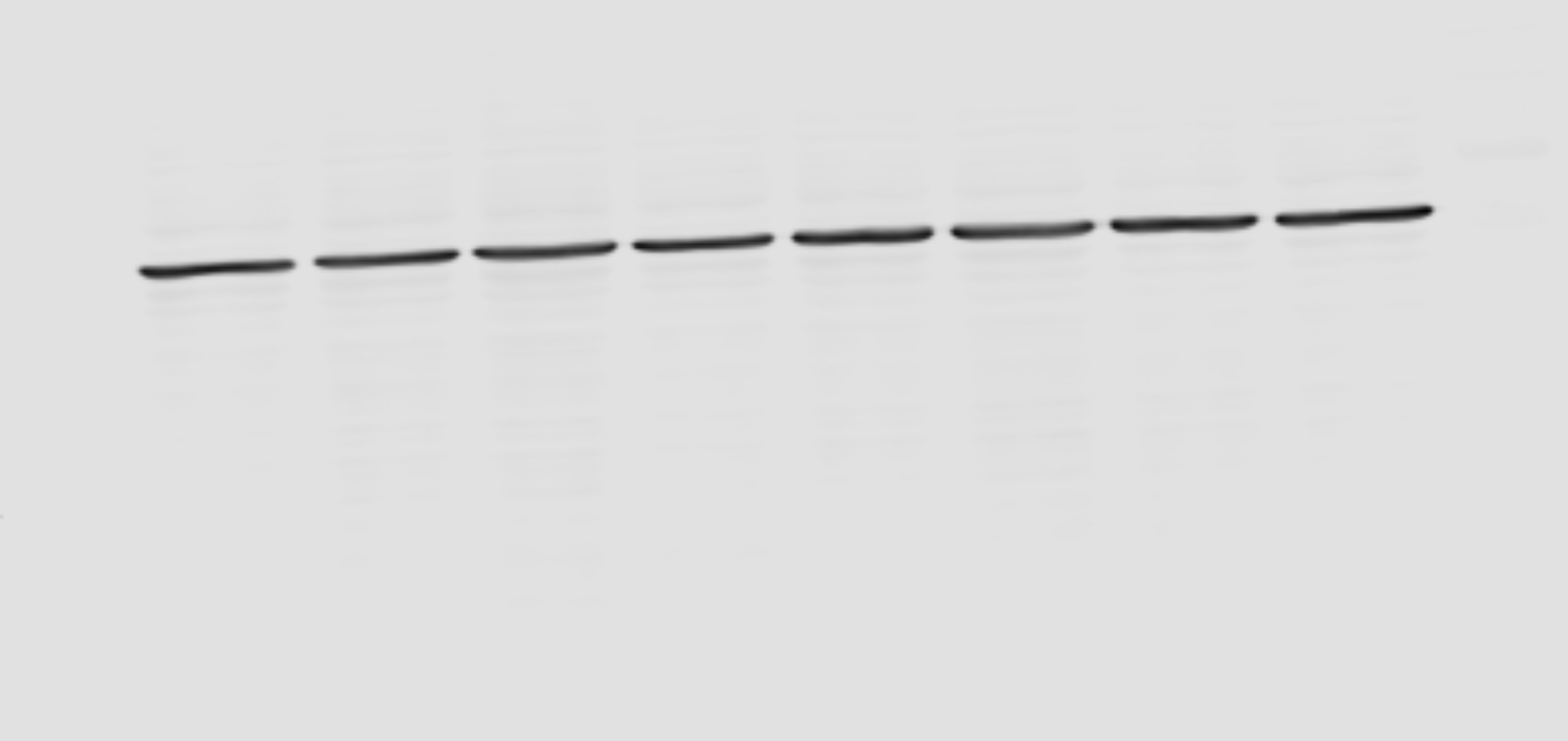

Supplement: Figure 6—source data 3. [file elife-103705-fig6-data3.zip › Figure 6- source data 2-Original files for western blot analysis displayed in Figure 6i/Original files for western blot analysis displayed in Figure 6i 4.tif]

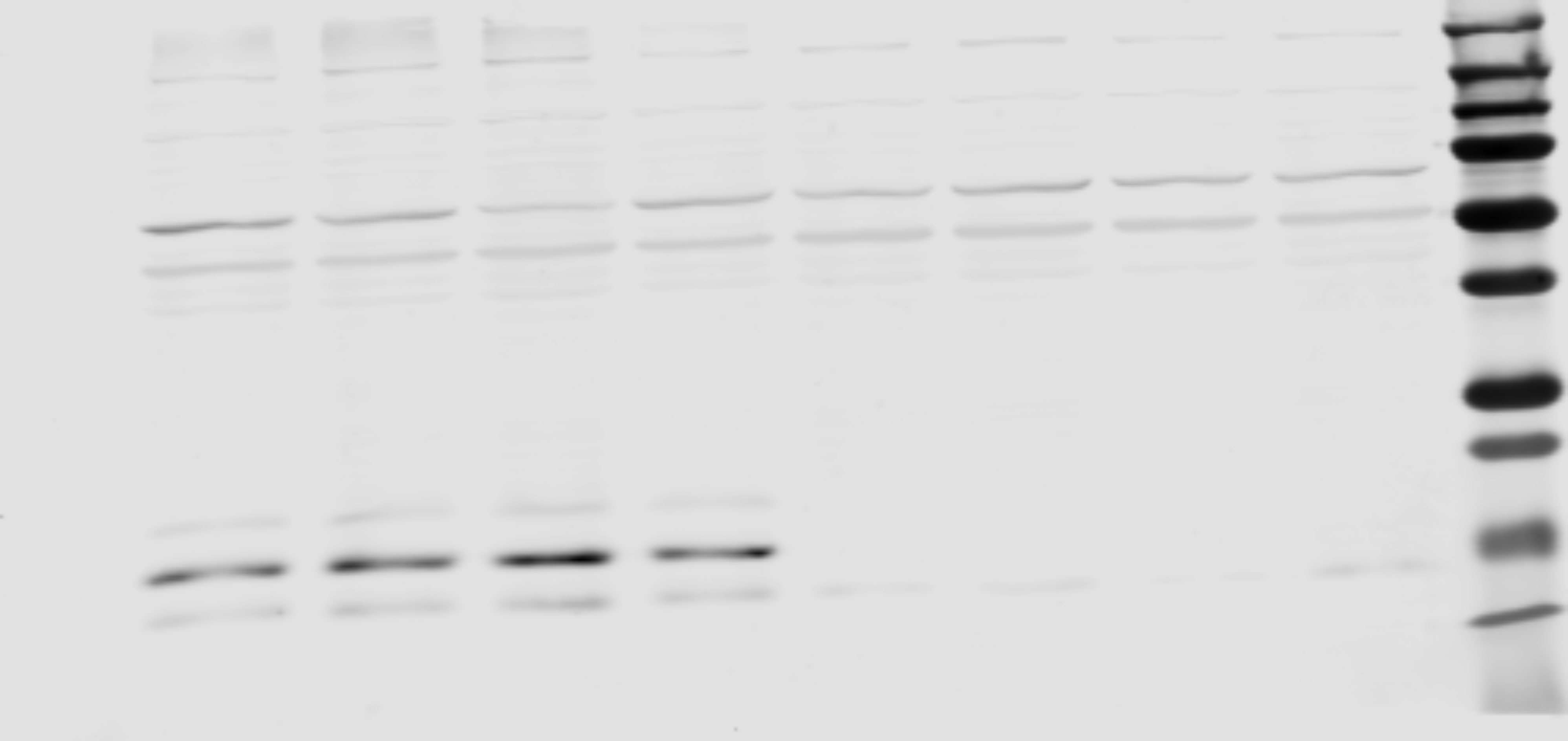

Supplement: Figure 6—source data 3. [file elife-103705-fig6-data3.zip › Figure 6- source data 2-Original files for western blot analysis displayed in Figure 6i/Original files for western blot analysis displayed in Figure 6i 5.tif]

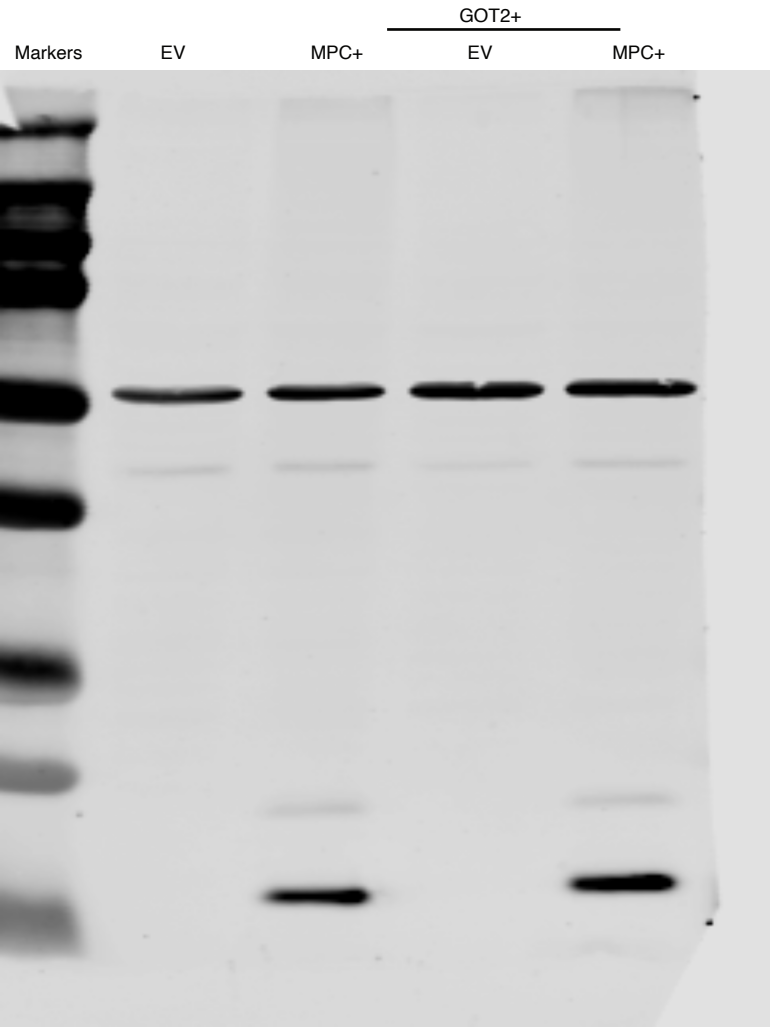

Supplement: Figure 6—figure supplement 1—source data 2. [file elife-103705-fig6-figsupp1-data2.zip › Figure 6-figure supplement 1k- PDF files containing originall western blots for Figure 6-figure supplement 1k, indicating the relevant bands and treatments./Figure 6-figure supplement 1k- PDF files containing originall western blots for Figure 6-figure supplement 1k, indicating the relevant bands an]

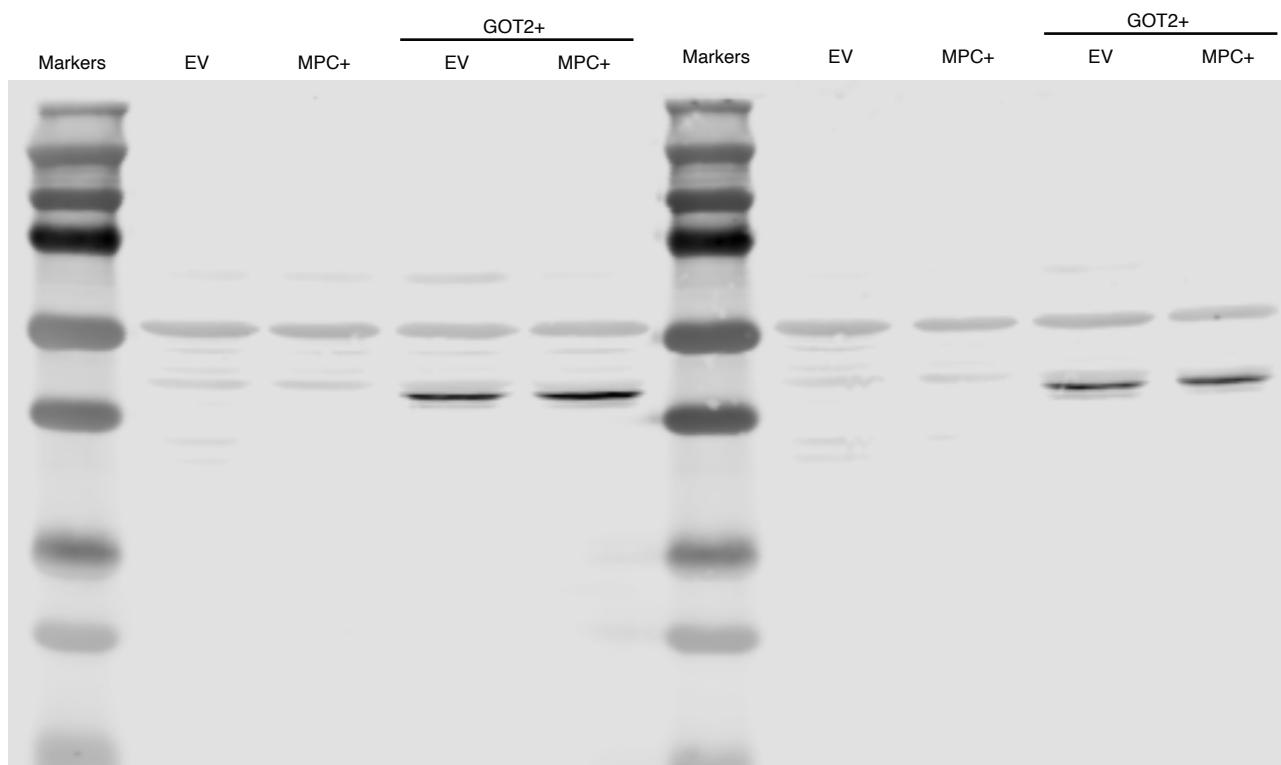

Supplement: Figure 6—figure supplement 1—source data 2. [file elife-103705-fig6-figsupp1-data2.zip › Figure 6-figure supplement 1k- PDF files containing originall western blots for Figure 6-figure supplement 1k, indicating the relevant bands and treatments./Figure 6-figure supplement 1k- PDF files containing originall western blots for Figure 6-figure supplement 1k, indicating the relevant bands an]

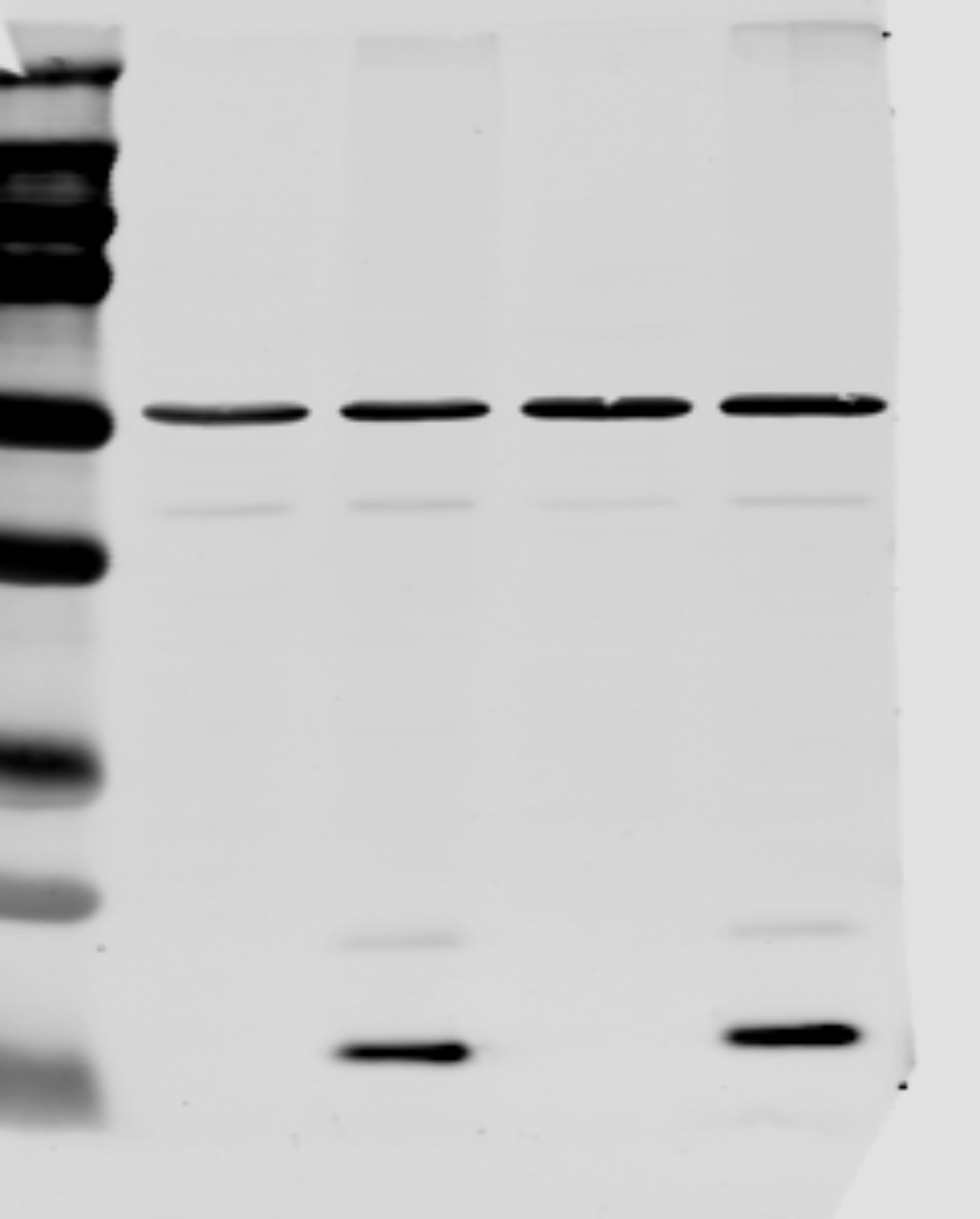

Supplement: Figure 6—figure supplement 1—source data 3. [file elife-103705-fig6-figsupp1-data3.zip › Original files for western blot analysis displayed in Figure 6-figure supplement 1k/Original files for western blot analysis displayed in Figure 6-figure supplement 1k 2.tif]

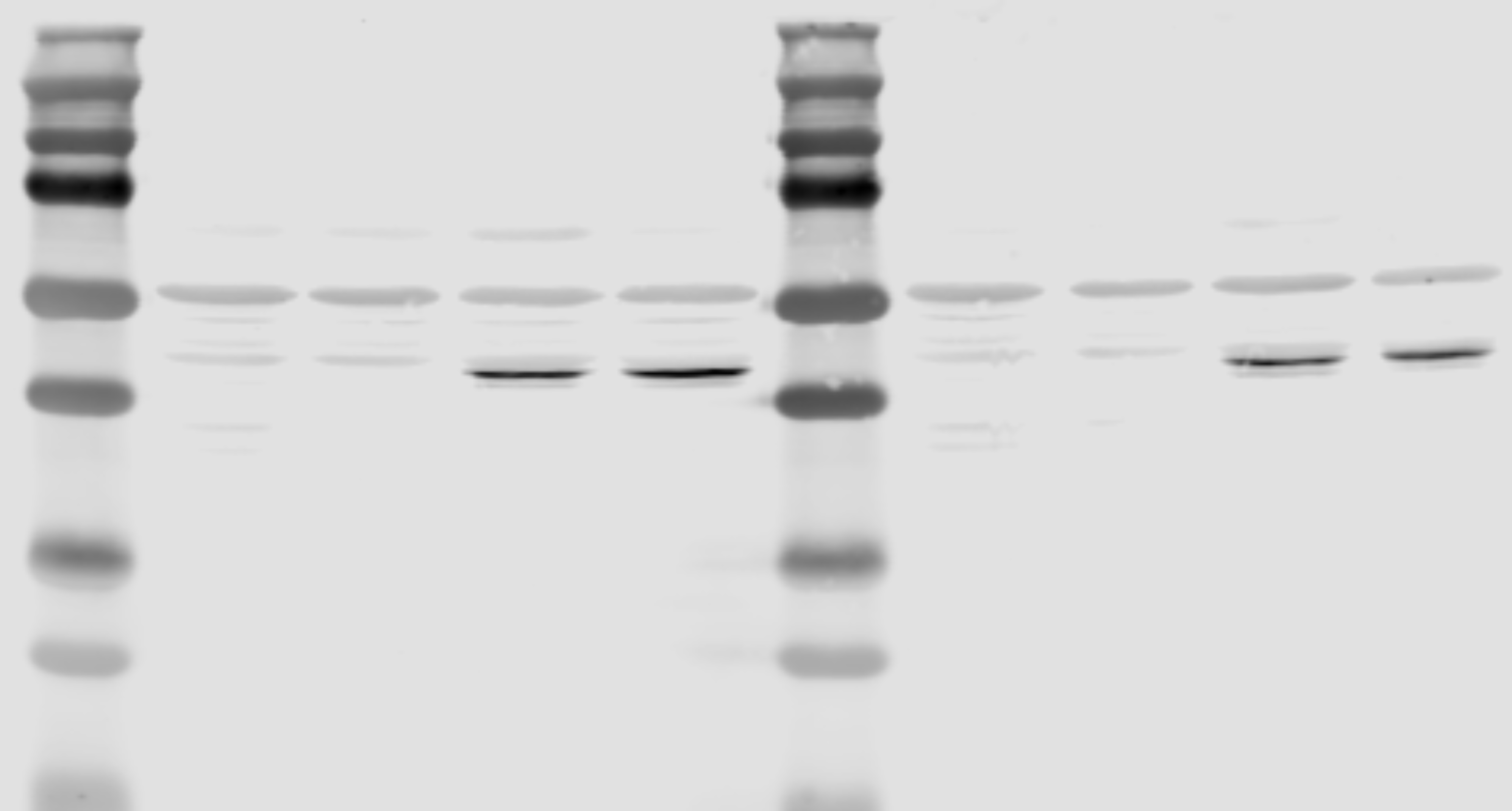

Supplement: Figure 6—figure supplement 1—source data 3. [file elife-103705-fig6-figsupp1-data3.zip › Original files for western blot analysis displayed in Figure 6-figure supplement 1k/Original files for western blot analysis displayed in Figure 6-figure supplement 1k 1.tif]

GOT2ga

GOT2gb

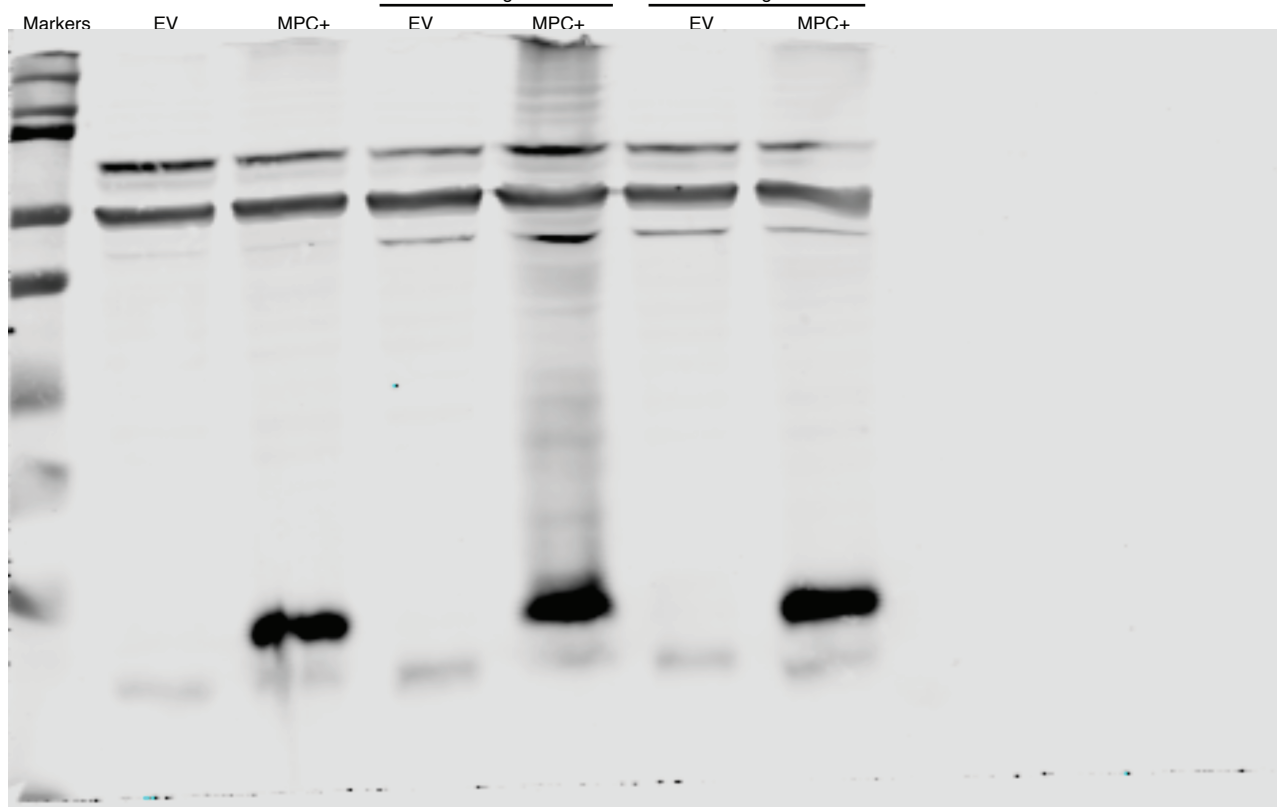

Supplement: Figure 6—figure supplement 1—source data 4. [file elife-103705-fig6-figsupp1-data4.zip › Figure 6-figure supplement 1i- PDF files containing originall western blots for Figure 6-figure supplement 1i, indicating the relevant bands and treatments./Figure 6-figure supplement 1i 2- PDF files containing originall western blots for Figure 6-figure supplement 1i, indicating the relevant bands ]

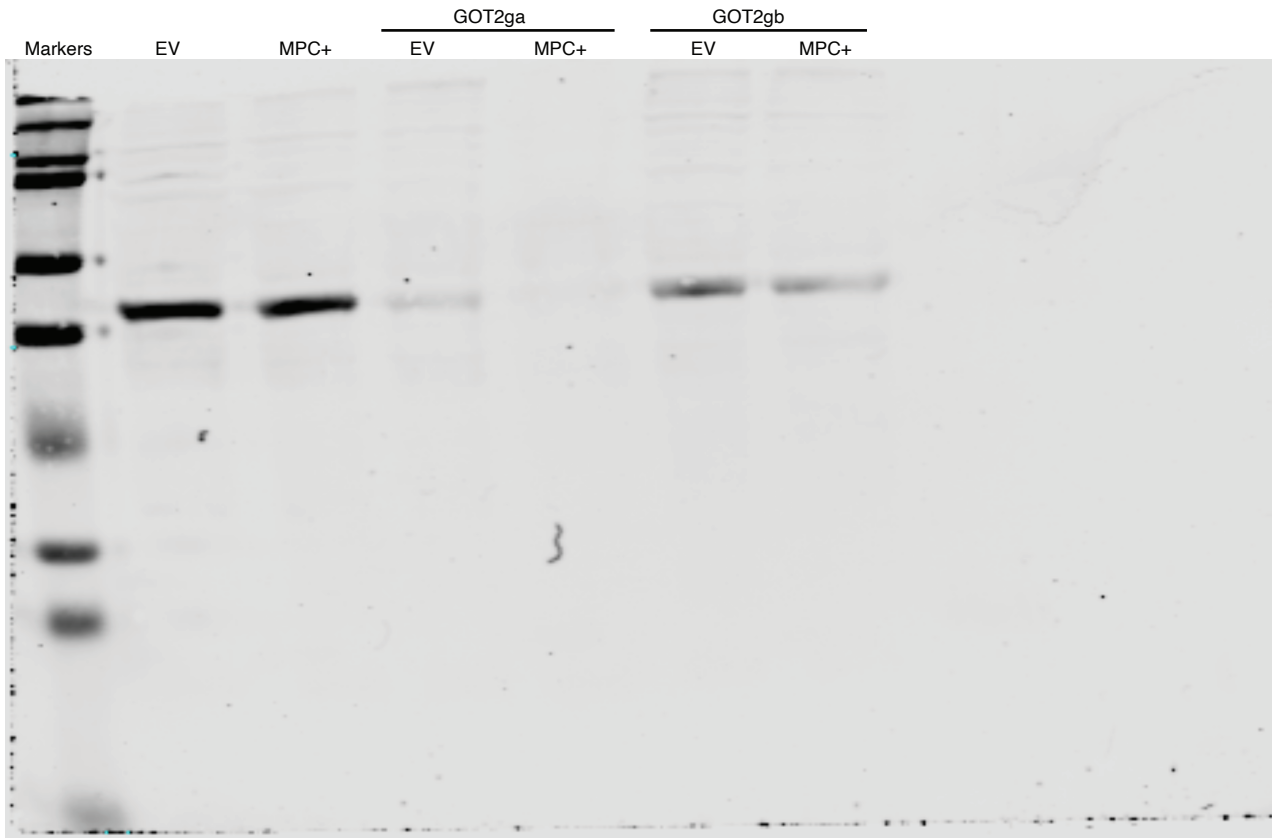

Supplement: Figure 6—figure supplement 1—source data 4. [file elife-103705-fig6-figsupp1-data4.zip › Figure 6-figure supplement 1i- PDF files containing originall western blots for Figure 6-figure supplement 1i, indicating the relevant bands and treatments./Figure 6-figure supplement 1i 1- PDF files containing originall western blots for Figure 6-figure supplement 1i, indicating the relevant bands ]

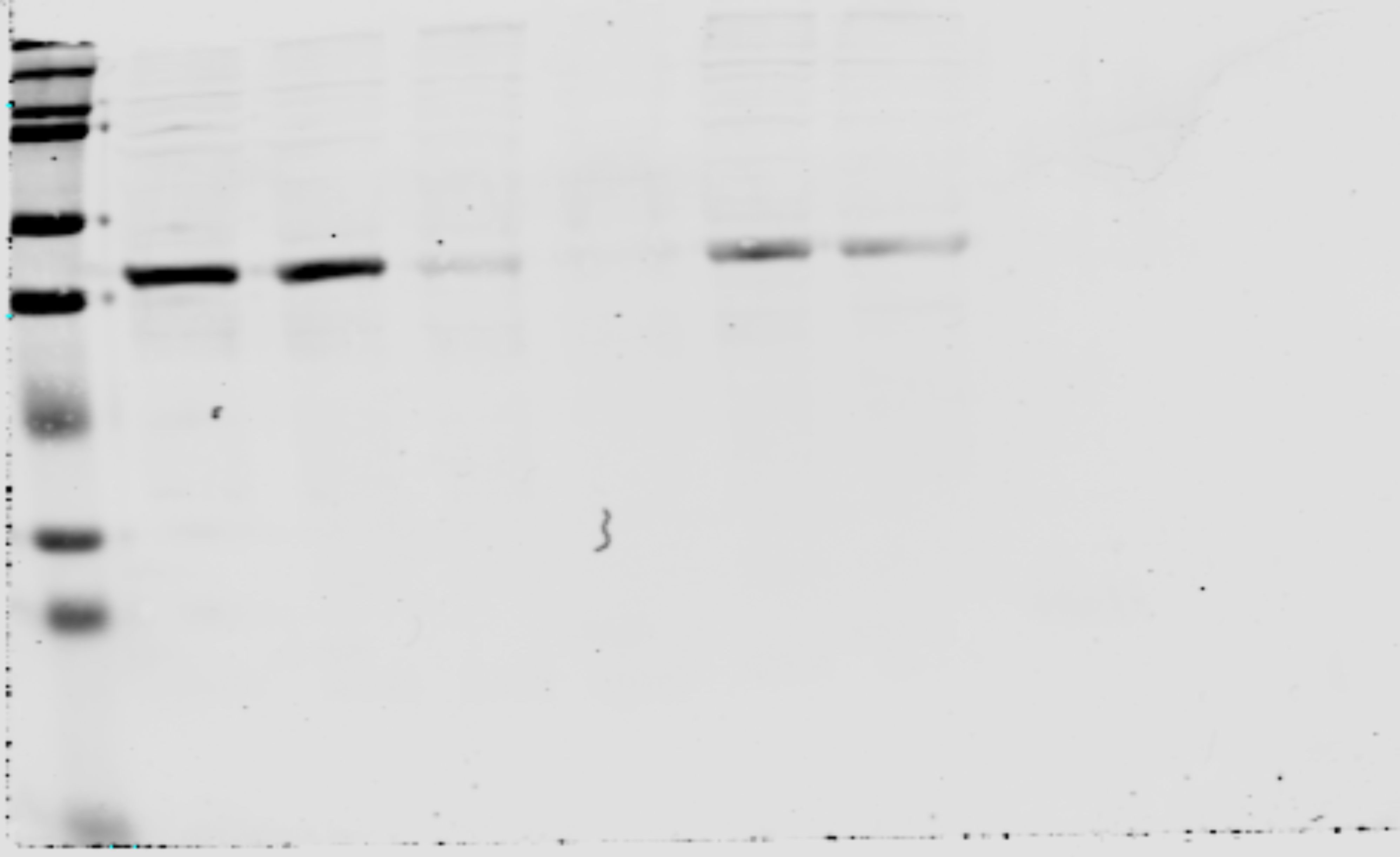

Supplement: Figure 6—figure supplement 1—source data 5. [file elife-103705-fig6-figsupp1-data5.zip › Original files for western blot analysis displayed in Figure 6-figure supplement 1i/Original files for western blot analysis displayed in Figure 6-figure supplement 1i 1.tif]

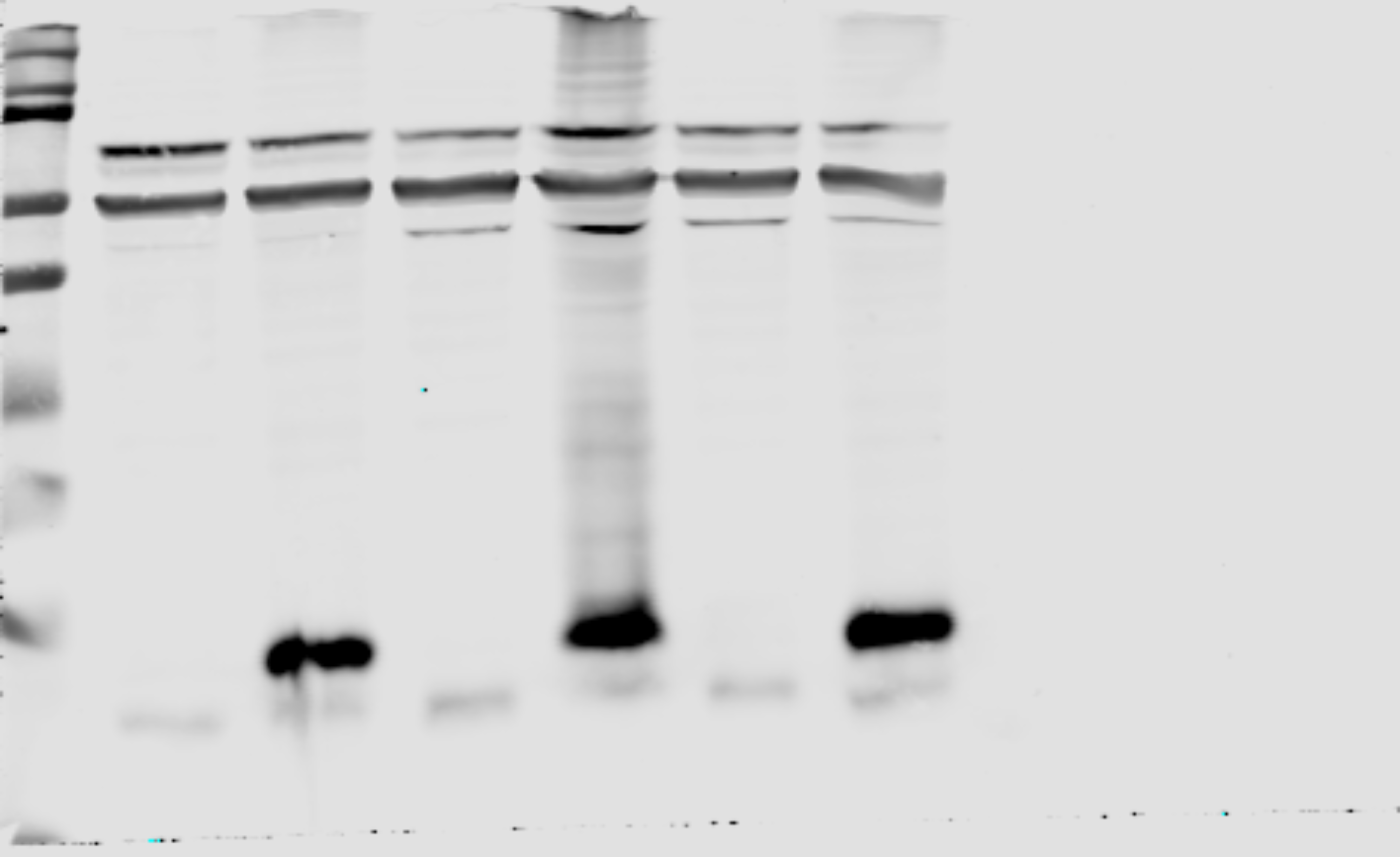

Supplement: Figure 6—figure supplement 1—source data 5. [file elife-103705-fig6-figsupp1-data5.zip › Original files for western blot analysis displayed in Figure 6-figure supplement 1i/Original files for western blot analysis displayed in Figure 6-figure supplement 1i 2.tif]

Marker

Hepatocyte+GFP

Hepatocyte+MPC1/2

HepG2

HepG2+MPC1/2

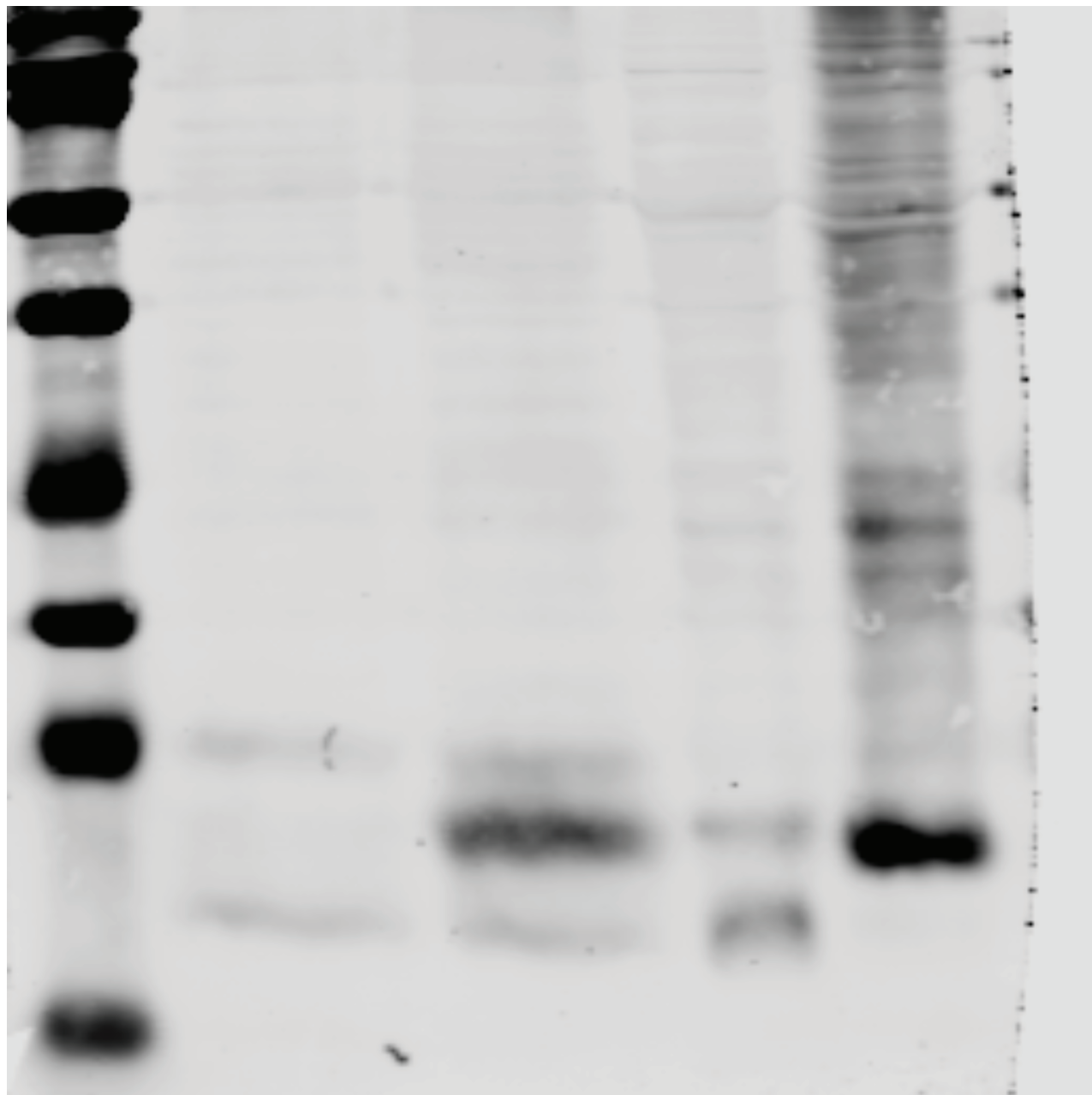

MPC1

Supplement: Figure 7—source data 2. [file elife-103705-fig7-data2.zip › Figure 7-source data 1- PDF files containing originall western blots for Figure 7a,indicating the relevant bands and treatments./Figure 7-source data 1- PDF files containing originall western blots for Figure 7a,indicating the relevant bands and treatments 2.pdf]

Marker      Hepatocyte+GFP      Hepatocyte+MPC1/2      HepG2      HepG2+MPC1/2

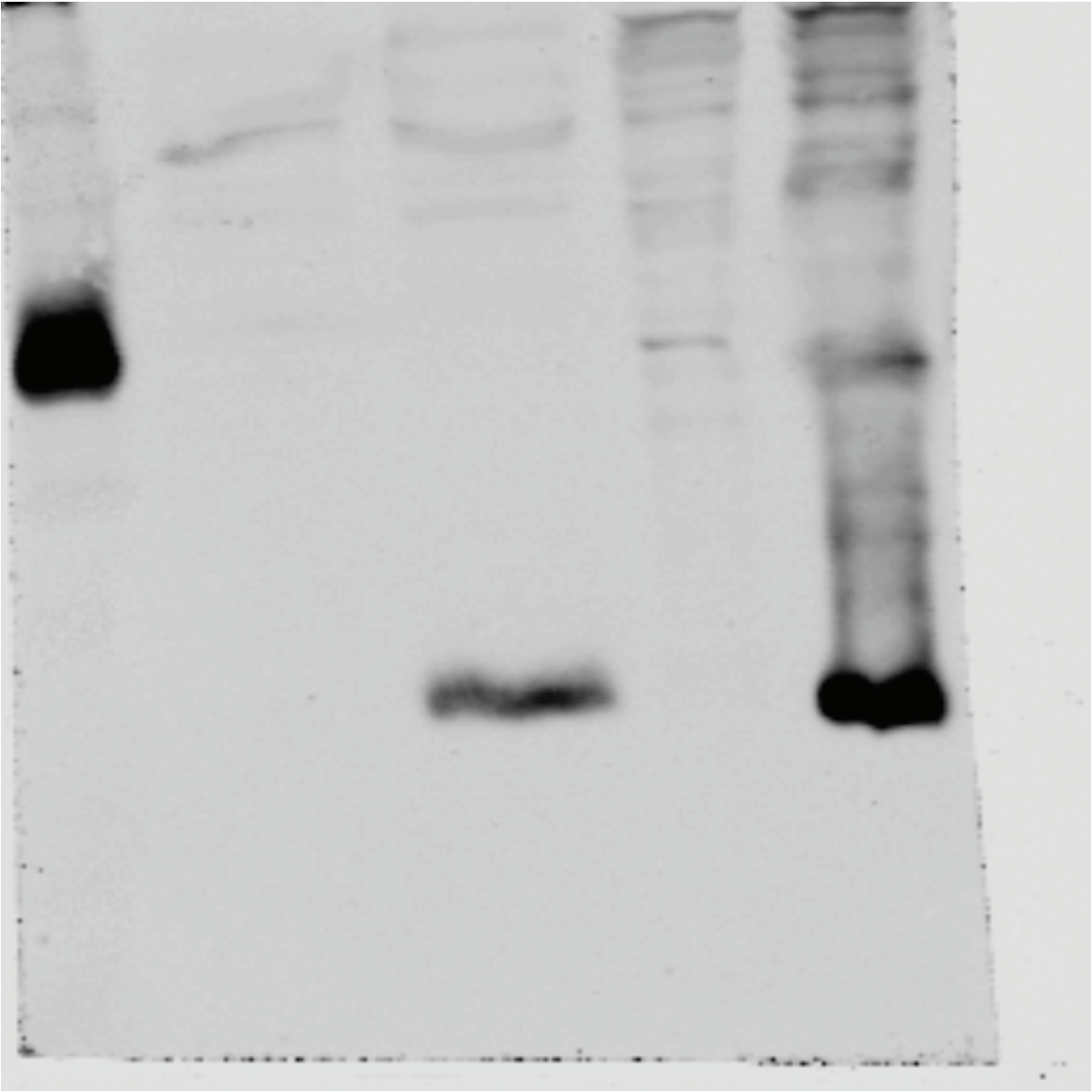

Flag

Supplement: Figure 7—source data 2. [file elife-103705-fig7-data2.zip › Figure 7-source data 1- PDF files containing originall western blots for Figure 7a,indicating the relevant bands and treatments./Figure 7-source data 1- PDF files containing originall western blots for Figure 7a,indicating the relevant bands and treatments 3.pdf]

Marker

Hepatocyte+GFP

Hepatocyte+MPC1/2

HepG2

HepG2+MPC1/2

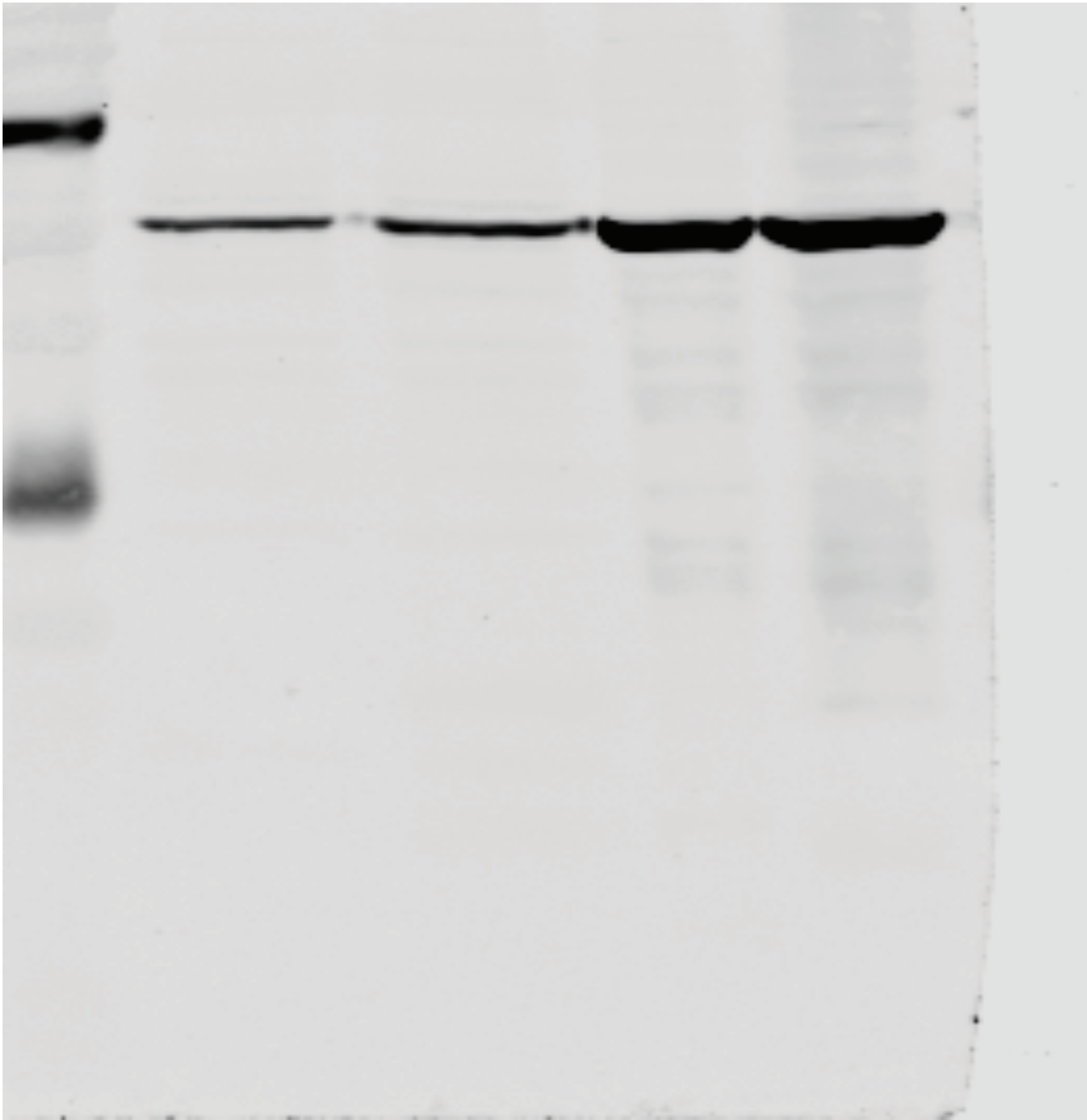

Tubulin

Supplement: Figure 7—source data 2. [file elife-103705-fig7-data2.zip › Figure 7-source data 1- PDF files containing originall western blots for Figure 7a,indicating the relevant bands and treatments./Figure 7-source data 1- PDF files containing originall western blots for Figure 7a,indicating the relevant bands and treatments 6.pdf]

Marker

Hepatocyte+GFP

Hepatocyte+MPC1/2

HepG2

HepG2+MPC1/2

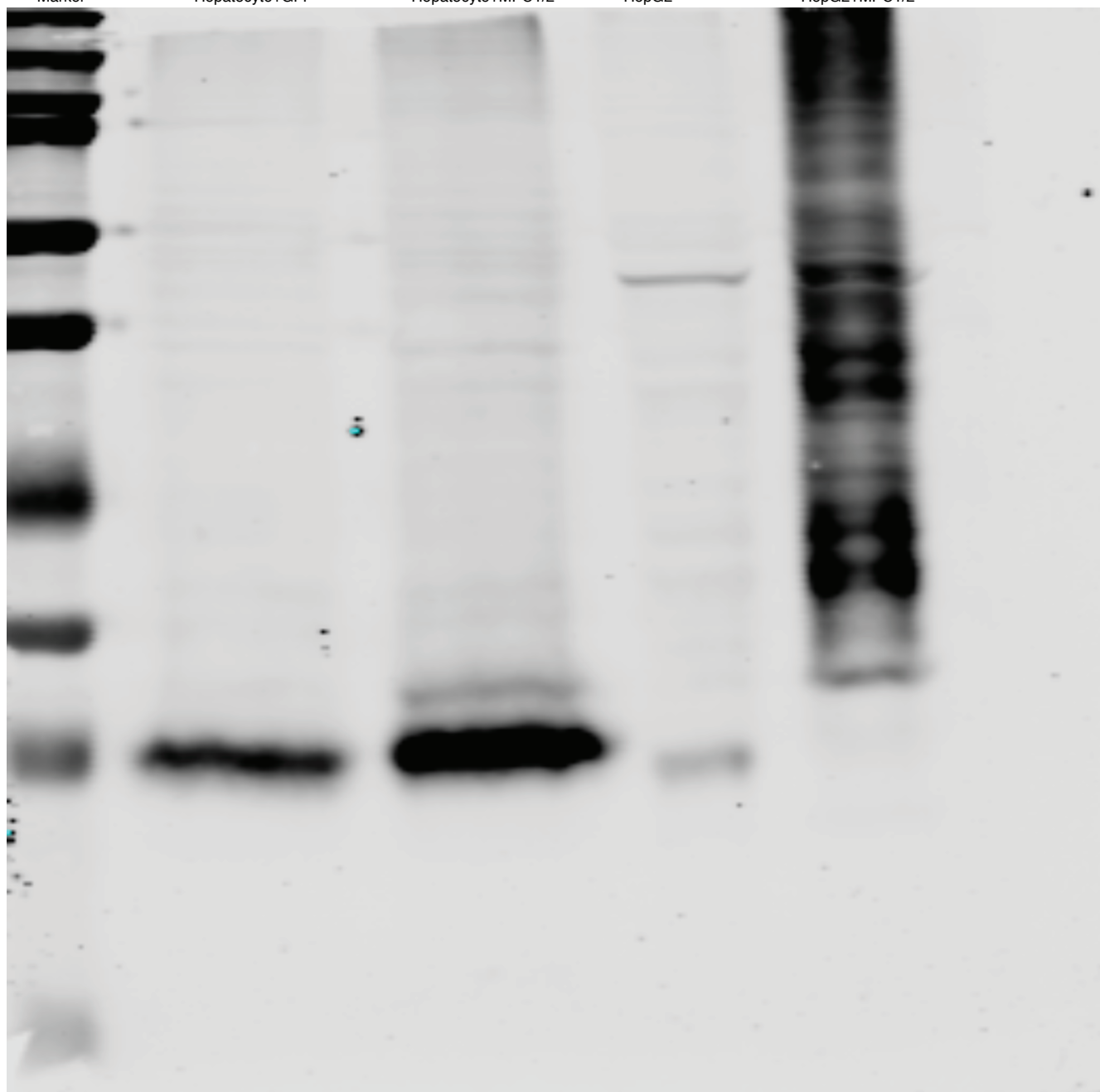

MPC2

Supplement: Figure 7—source data 2. [file elife-103705-fig7-data2.zip › Figure 7-source data 1- PDF files containing originall western blots for Figure 7a,indicating the relevant bands and treatments./Figure 7-source data 1- PDF files containing originall western blots for Figure 7a,indicating the relevant bands and treatments 4.pdf]

Marker

Hepatocyte+GFP

Hepatocyte+MPC1/2

HepG2

HepG2+MPC1/2

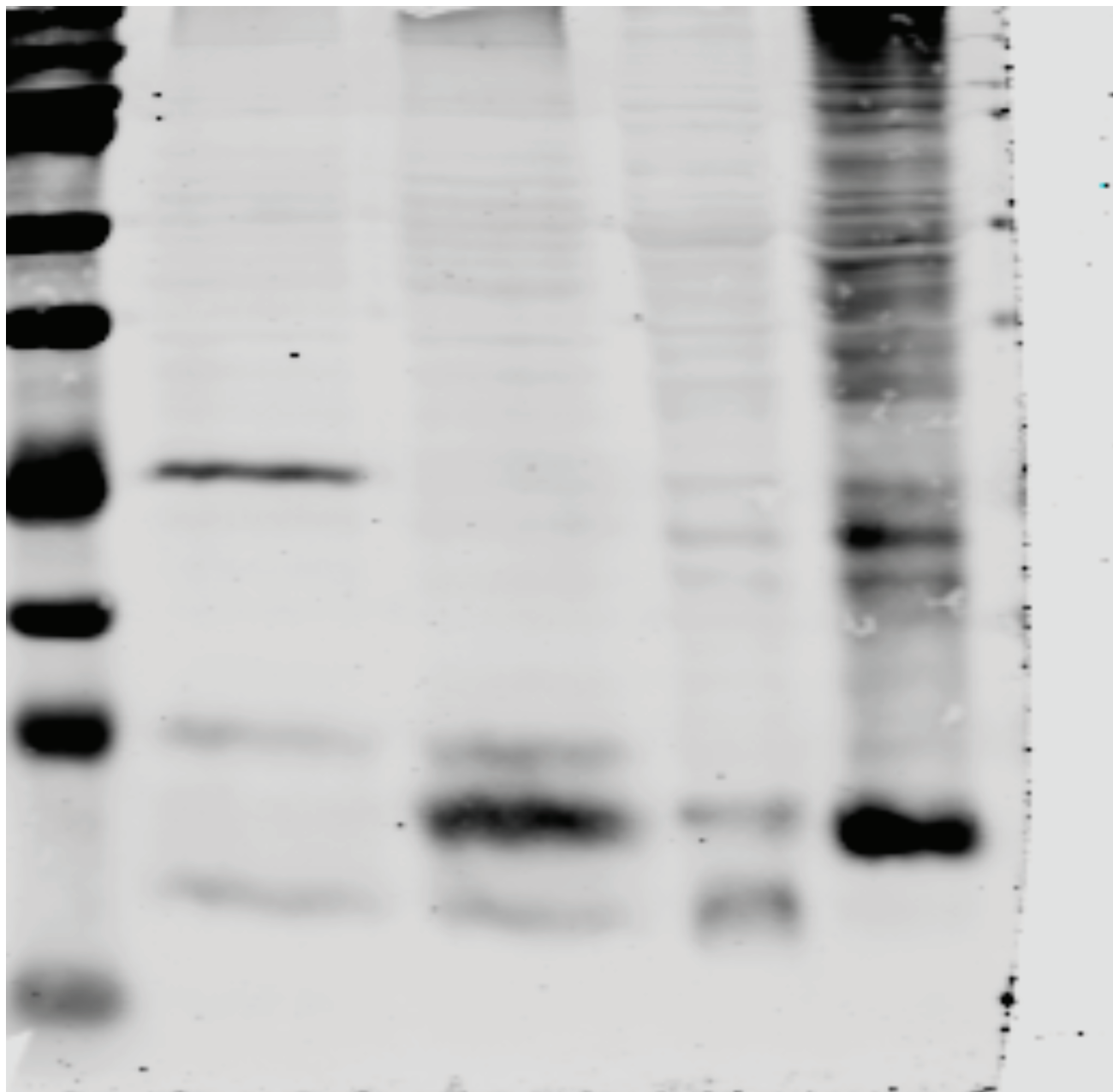

GFP

HA

Supplement: Figure 7—source data 2. [file elife-103705-fig7-data2.zip › Figure 7-source data 1- PDF files containing originall western blots for Figure 7a,indicating the relevant bands and treatments./Figure 7-source data 1- PDF files containing originall western blots for Figure 7a,indicating the relevant bands and treatments 5.pdf]

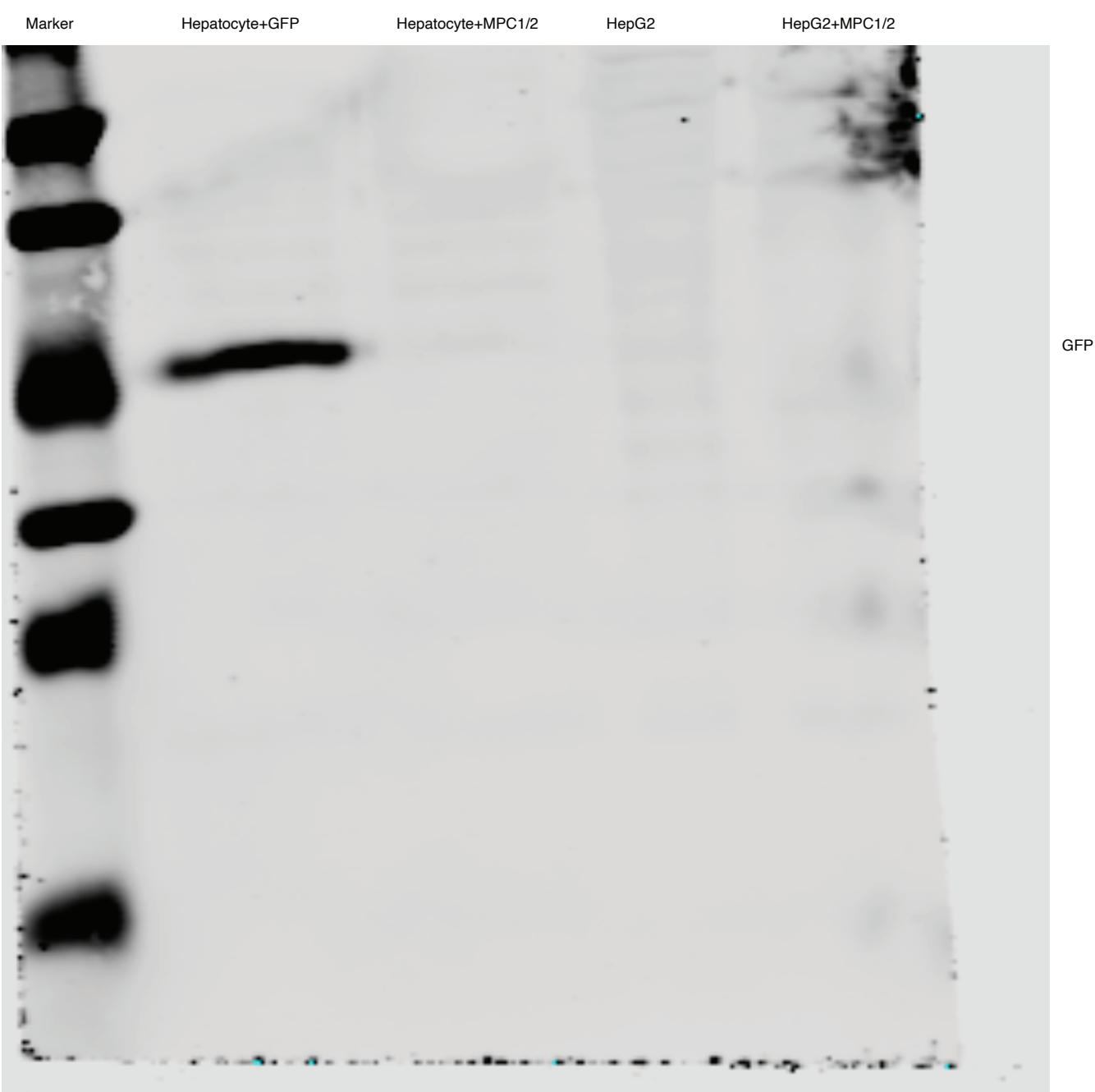

Supplement: Figure 7—source data 2. [file elife-103705-fig7-data2.zip › Figure 7-source data 1- PDF files containing originall western blots for Figure 7a,indicating the relevant bands and treatments./Figure 7-source data 1- PDF files containing originall western blots for Figure 7a,indicating the relevant bands and treatments 1..pdf]

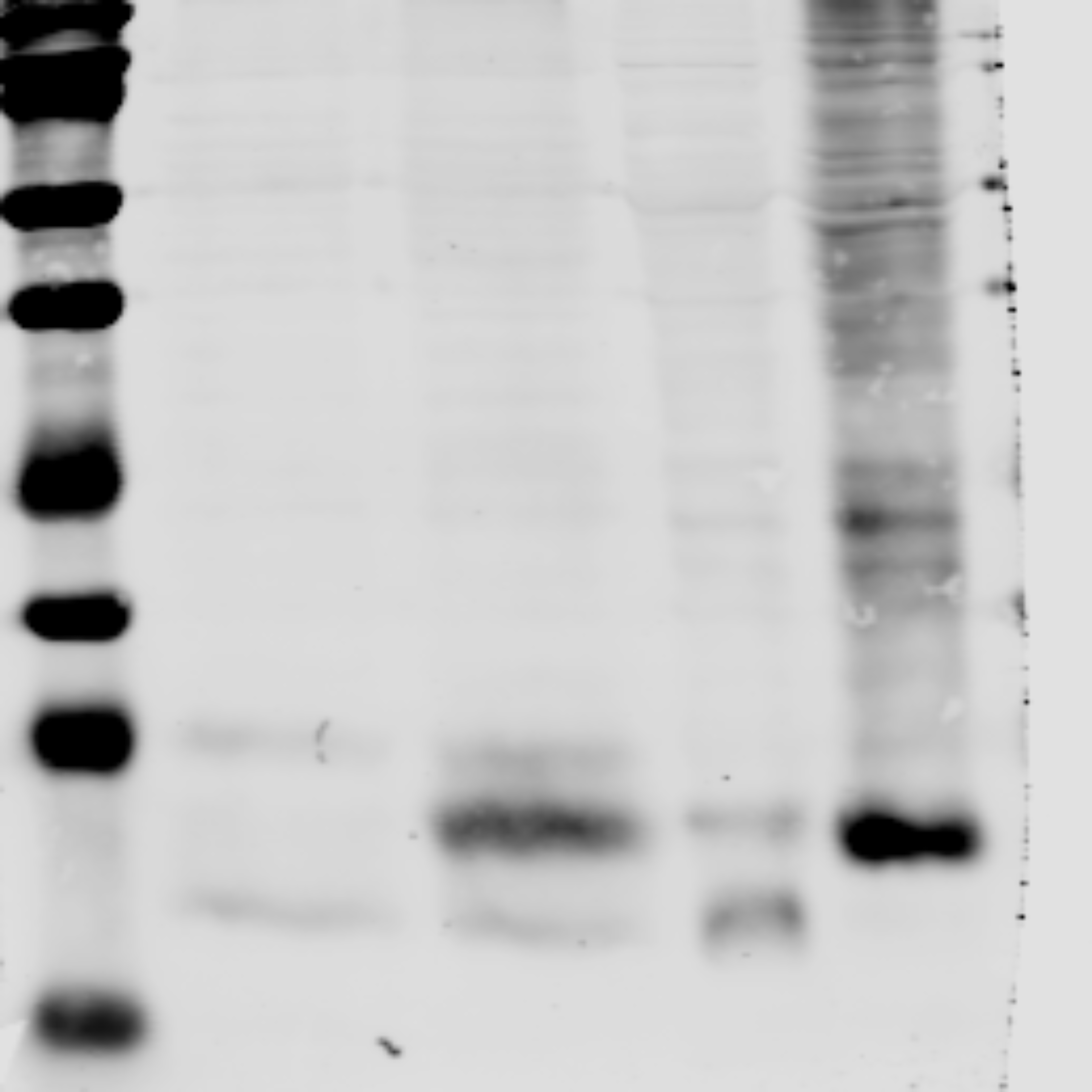

Supplement: Figure 7—source data 3. [file elife-103705-fig7-data3.zip › Figure 7- source data 2-Original files for western blot analysis displayed in Figure 7a I/Original files for western blot analysis displayed in Figure 7a 2.tif]

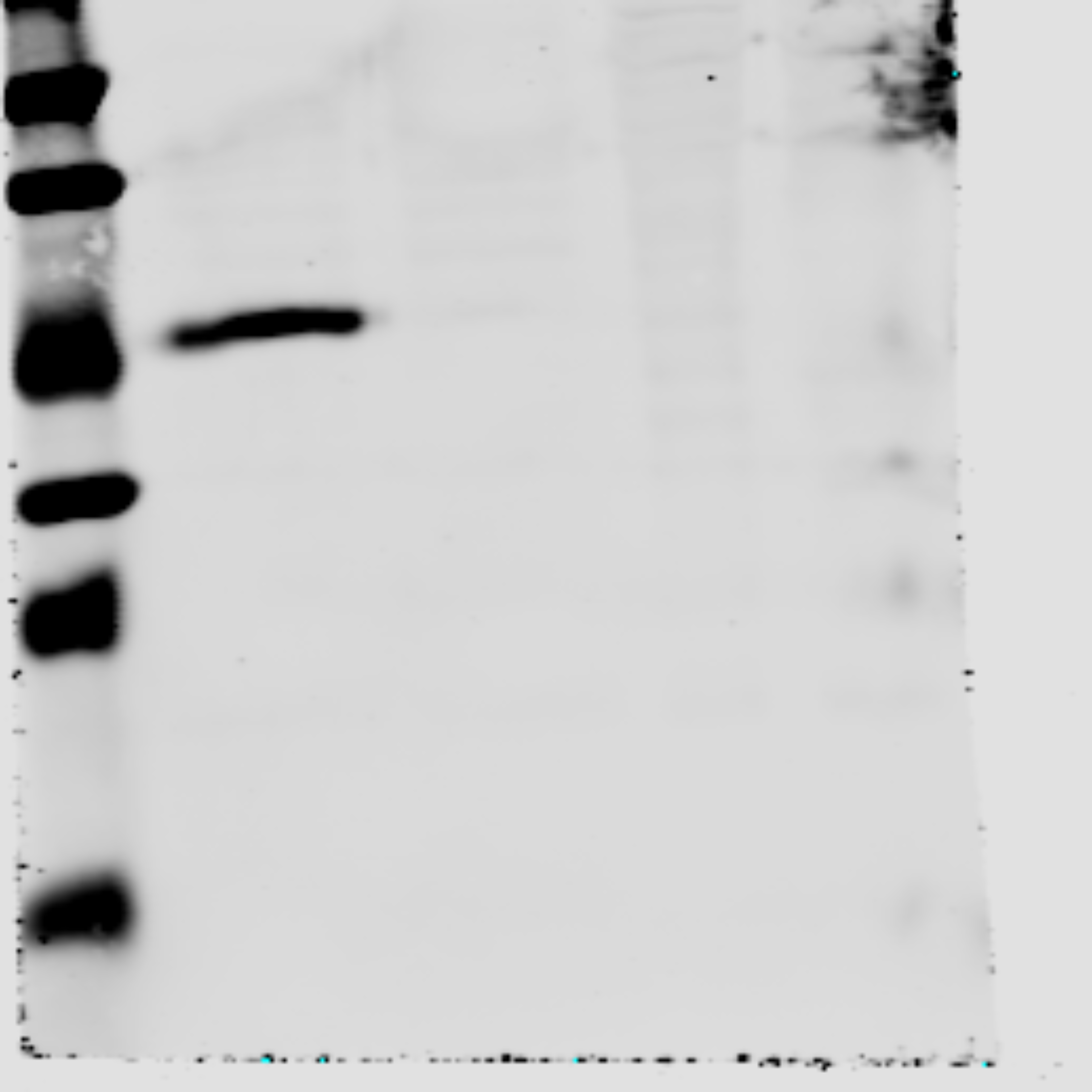

Supplement: Figure 7—source data 3. [file elife-103705-fig7-data3.zip › Figure 7- source data 2-Original files for western blot analysis displayed in Figure 7a I/Original files for western blot analysis displayed in Figure 7a 1.tif]

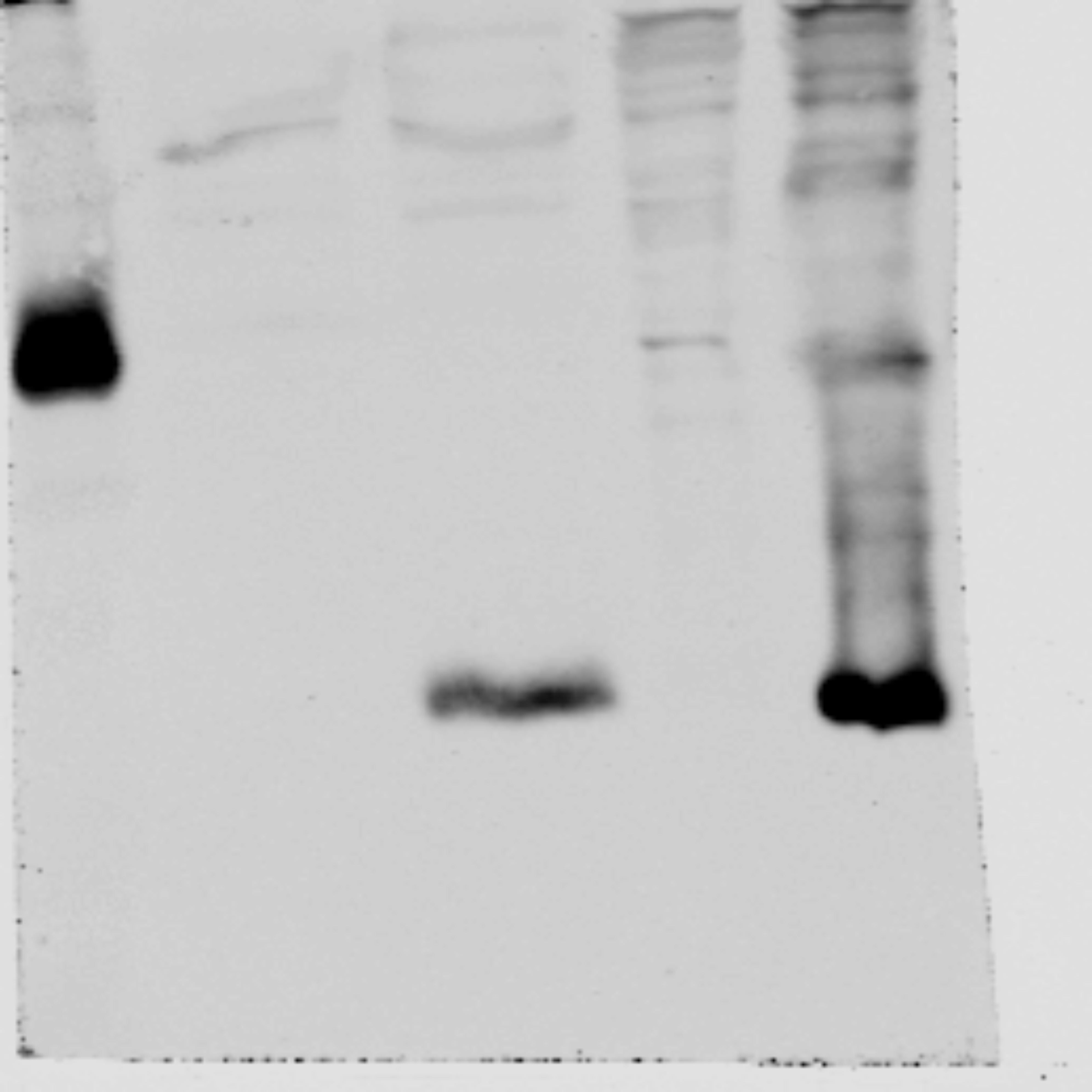

Supplement: Figure 7—source data 4. [file elife-103705-fig7-data4.zip › Figure 7- source data 3-Original files for western blot analysis displayed in Figure 7a II/Original files for western blot analysis displayed in Figure 7a 3.tif]

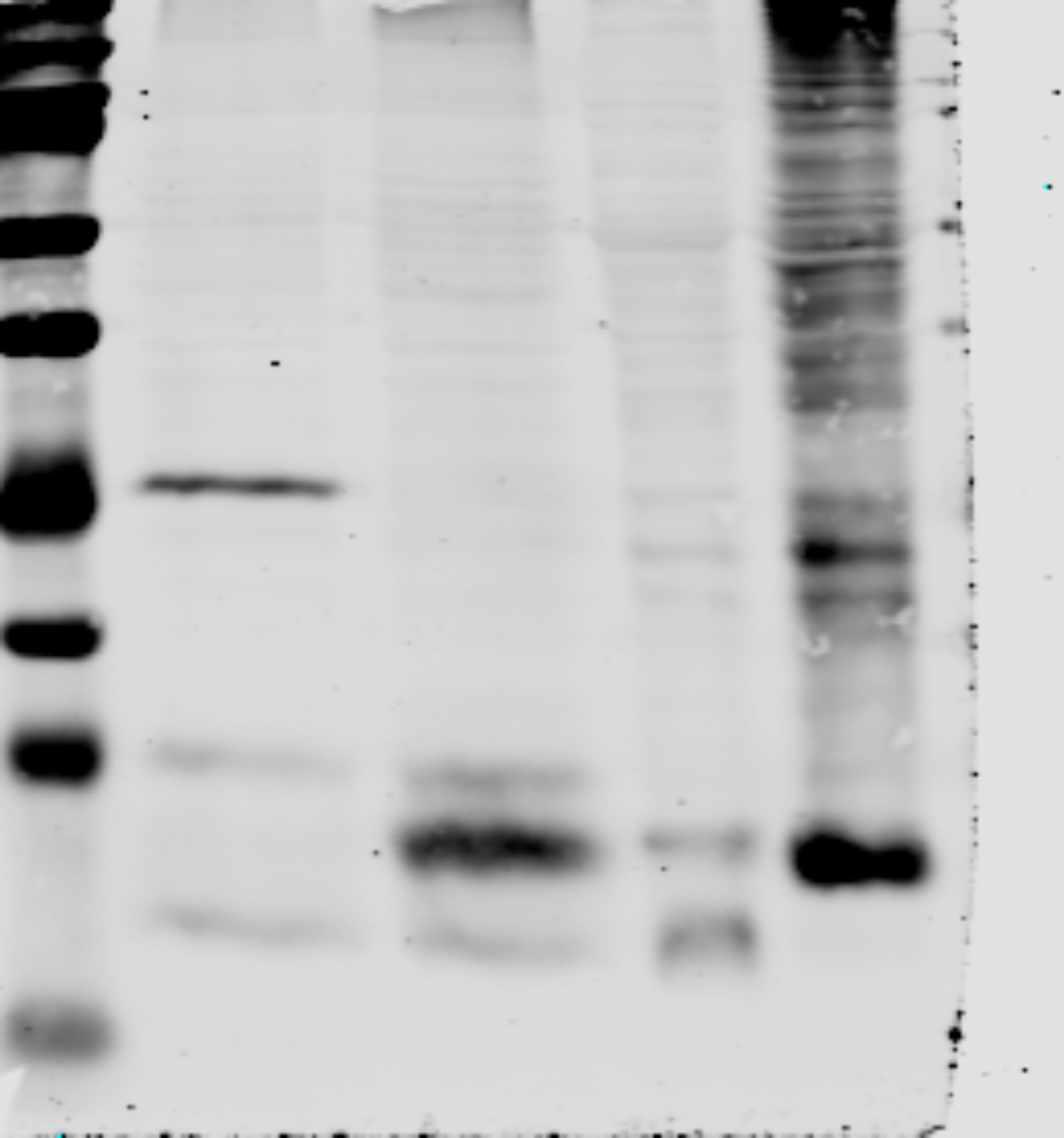

Supplement: Figure 7—source data 4. [file elife-103705-fig7-data4.zip › Figure 7- source data 3-Original files for western blot analysis displayed in Figure 7a II/Original files for western blot analysis displayed in Figure 7a 4.tif]

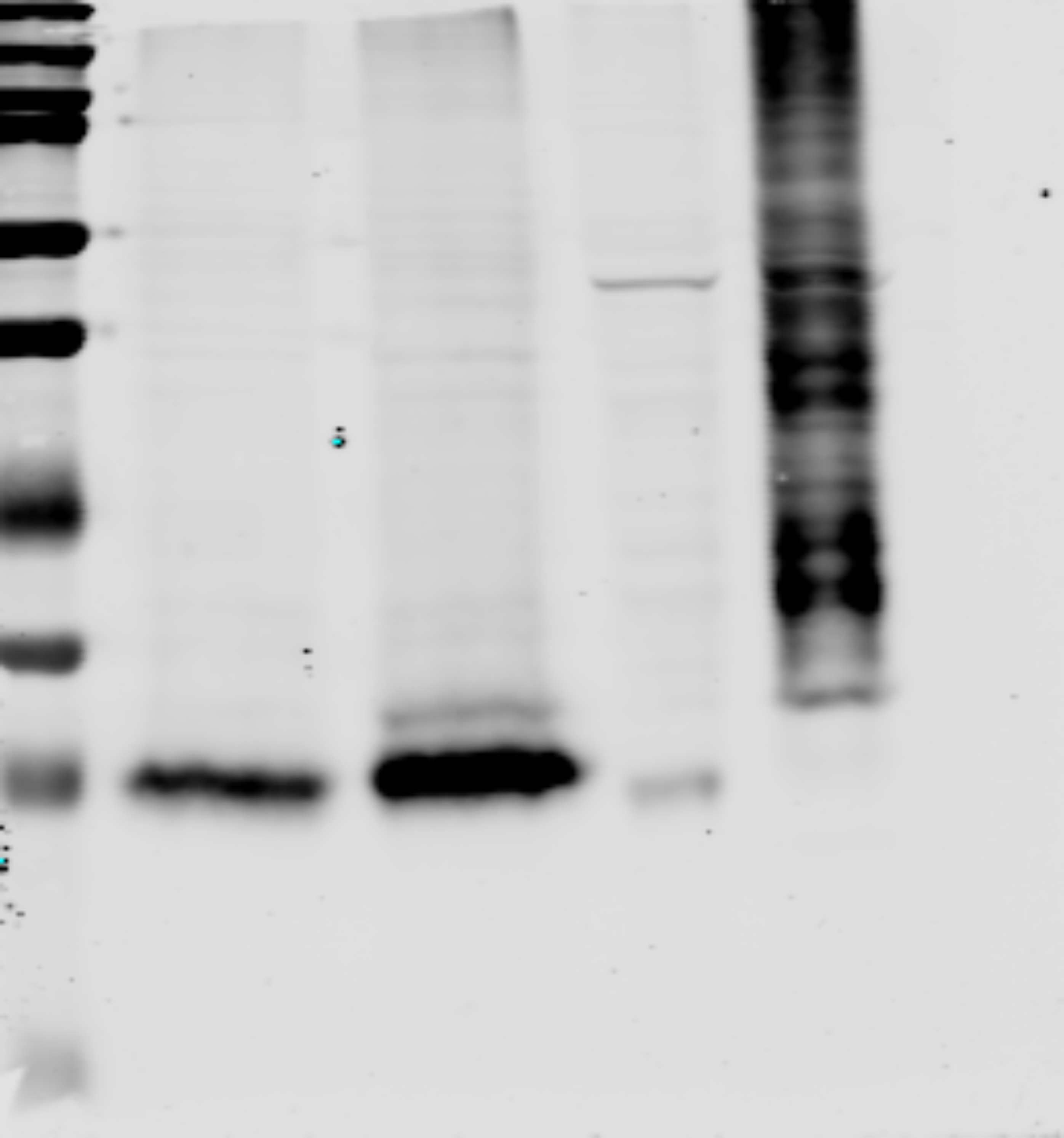

Supplement: Figure 7—source data 5. [file elife-103705-fig7-data5.zip › Figure 7- source data 4- Original files for western blot analysis displayed in Figure 7a III/Original files for western blot analysis displayed in Figure 7a 5.tif]

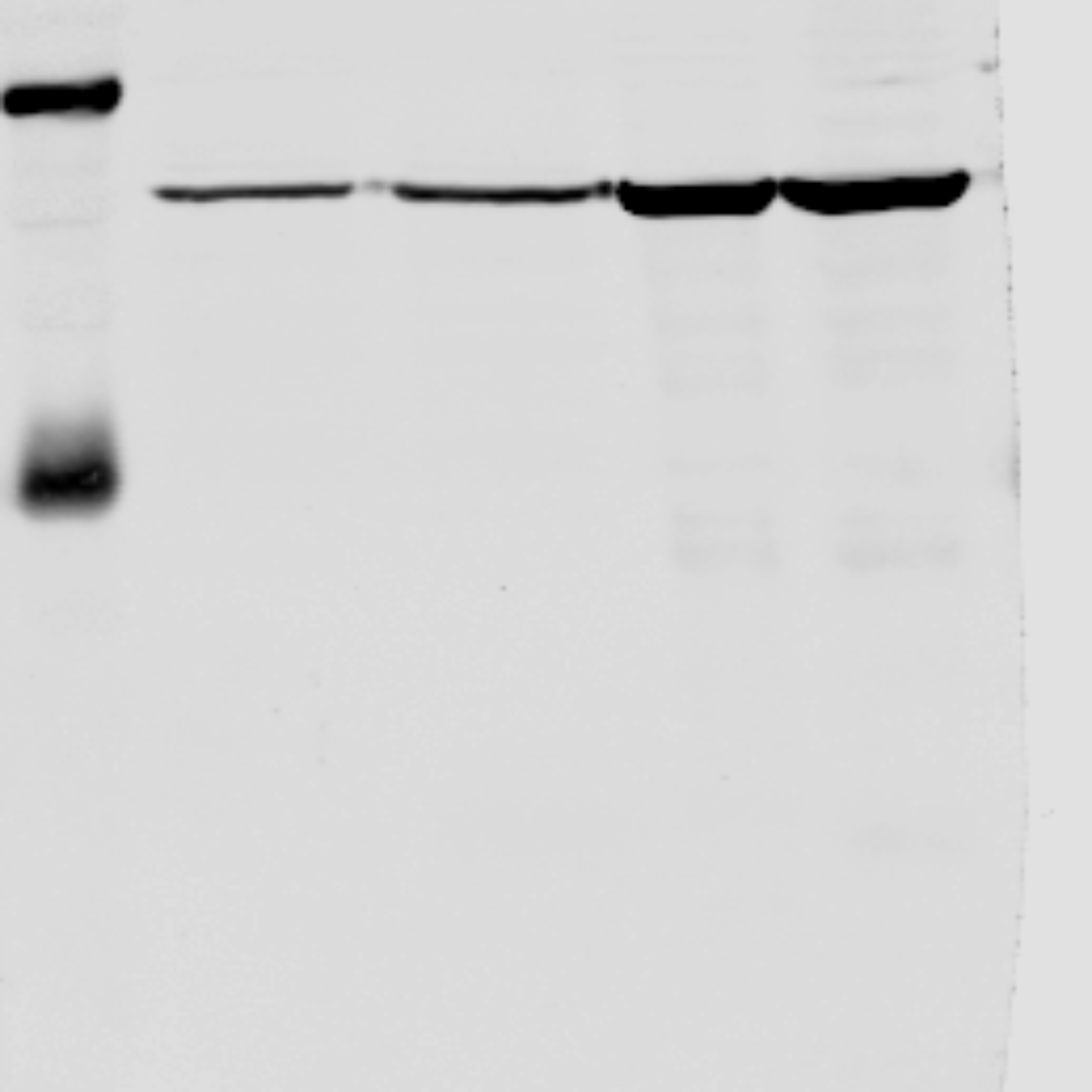

Supplement: Figure 7—source data 5. [file elife-103705-fig7-data5.zip › Figure 7- source data 4- Original files for western blot analysis displayed in Figure 7a III/Original files for western blot analysis displayed in Figure 7a 6.tif]
